# Insulated conjugated bimetallopolymer with sigmoidal response by dual self-controlling system as a biomimetic material

**DOI:** 10.1038/s41467-019-14271-2

**Published:** 2020-01-21

**Authors:** Hiroshi Masai, Takuya Yokoyama, Hiromichi V. Miyagishi, Maning Liu, Yasuhiro Tachibana, Tetsuaki Fujihara, Yasushi Tsuji, Jun Terao

**Affiliations:** 1 Department of Basic Science, Graduate School of Arts and Sciences, The University of Tokyo, Tokyo, 153-8902 Japan; 2 Department of Energy and Hydrocarbon Chemistry, Graduate School of Engineering, Kyoto University, Kyoto, 615-8510 Japan; 3 School of Engineering, RMIT University, Bundoora, Victoria, 3083 Australia

**Keywords:** Organometallic chemistry, Materials chemistry, Polymer chemistry, Supramolecular chemistry

## Abstract

Biological systems are known to spontaneously adjust the functioning of neurotransmitters, ion channels, and the immune system, being promoted or regulated through allosteric effects or inhibitors, affording non-linear responses to external stimuli. Here we report that an insulated conjugated bimetallopolymer, in which Ru(II) and Pt(II) complexes are mutually connected with insulated conjugations, exhibits phosphorescence in response to CO gas. The net profile corresponds to a sigmoidal response with a dual self-controlling system, where drastic changes were exhibited at two threshold concentrations. The first threshold for activation of the system is triggered by the depolymerization of the non-radiative conjugated polymer to luminescent monomers, while the second one for regulation is triggered by the switch in the rate-determining step of the Ru complex. Such a molecular design with cooperative multiple transition metals would provide routes for the development of higher-ordered artificial molecular systems bearing bioinspired responses with autonomous modulation.

## Abstract (web summary)

Molecules can serve as biomimetic sensors, but dual self-controlling systems that involve self-activation and self-regulation are difficult to mimic. Here the authors report an insulated conjugated bimetallopolymer with cooperative multiple transition metals that phosphoresces in response to carbon monoxide.

## Introduction

**Fig. 1:**
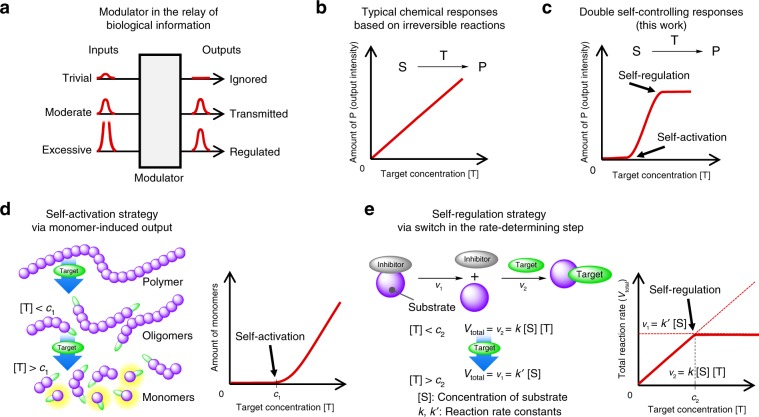
Conceptual illustration of sigmoidal response by dual self-controlling system. **a** Modulator in the relay of biological information. **b** Chemical responses for the target concentration in conventional responses. **c** Dual self-controlling responses (this work) (S: substrate, T: target, and P: product). **d** Schematic strategies for self-activation based on random depolymerization. **e** Self-regulation based on a switch in the rate-determining step (*c*_1_ and *c*_2_ are the threshold concentrations).

Natural systems can autonomously control their responses to promote and regulate various pathways, depending on the intensities of external stimuli. The controlling system plays an important role as a modulator in the relay of biological information and prevents needless responses to trivial and excessive inputs (Fig. 1a)^1^. The natural self-controlling systems, i.e., self-activating and self-regulatory systems are attributed to the allosteric effects and cascade mechanisms, including reversible and irreversible reactions among multiple proteins and bioactive compounds^2,3^. The sigmoidal responses with multiple thresholds for inputs afford stable biological activities in the surrounding environment. For example, self-activating thresholds ignore weak stimuli and enhance the sensitivity above the thresholds, while self-regulatory thresholds suppress excess output signals toward the next system in the information relay. Such sophisticated mechanisms are essential in the functioning of neurotransmitters, immune systems, ion channels, and transport systems^4–6^. Several studies have proposed the molecular designs of biomimetic materials as sensors^7–10^, catalytic systems^11,12^, and machines transporting materials^13–15^. However, to the best of our knowledge, no artificial compound has been developed to mimic a dual self-controlling system that involves both self-activation and self-regulation to date. Mimicking such self-controlling systems has been regarded as a challenge, since such systems would provide autonomous modulations into diverse artificial materials and systems, such as sensors, catalysts, and machines, without any human control.

As opposed to reversible reactions, chemical responses based on irreversible reactions have been utilized for memorizing the detection and for the directional transfer of information^16^. However, the responses (e.g., emission intensity) increase monotonically only when the target concentration [T] is not in excess (Fig. 1b). The artificial incorporation of multiple modulations into the irreversible reactions would be complex, despite their potential applications in the aforementioned biomimetics. In order to achieve a dual self-controlling system for [T] in irreversible reactions (Fig. 1c), herein, two strategies were applied on a single molecular component. The first strategy was a polymer to monomer conversion for self-activation at a low target concentration (Fig. 1d). The random depolymerization from polymer to monomer would initially afford oligomers and then monomers around a threshold concentration (*c*_1_), depending on the progress of the reaction. Accordingly, depolymerization to monomers should ultimately lead to the attainment of a threshold monomer concentration, which would correspond to the threshold concentration for activation via a monomer-induced output in a chemical reaction. The second strategy was targeted at self-regulation at high target concentrations, via a switch in the rate-determining step in reactions composed of two elementary steps, as observed in biological systems (Fig. 1e). The reaction was designed in such a way that the target was sensed after the intramolecular removal of the inhibitors, which prevented the substrates from reacting with the target. This was carried out to ensure that the dependence of the total reaction rate (*V*_total_) on [T] could be switched at the threshold concentration (*c*_2_), because the first reaction rate (*v*_1_) was independent of [T], while the other (*v*_2_) was dependent on it. Accordingly, the extent of the reaction would be regulated above *c*_2_ owing to the switch in the rate-determining step from *v*_2_ to *v*_1_. The compatibility of the two strategies on a single chemical compound with suitable concentration thresholds would afford unprecedented artificial sigmoidal responses in the aforementioned biomimetics with dual self-controlling systems. In this study, we demonstrate the artificial dual self-controlling responses using single polymer, which is composed of two transition metals and cyclodextrin-based insulated conjugation. The cooperative multiple transition metals provide excellent biomimetic response at two threshold concentrations of CO.

## Results

### Design for dual self-controlling system

**Fig. 2:**
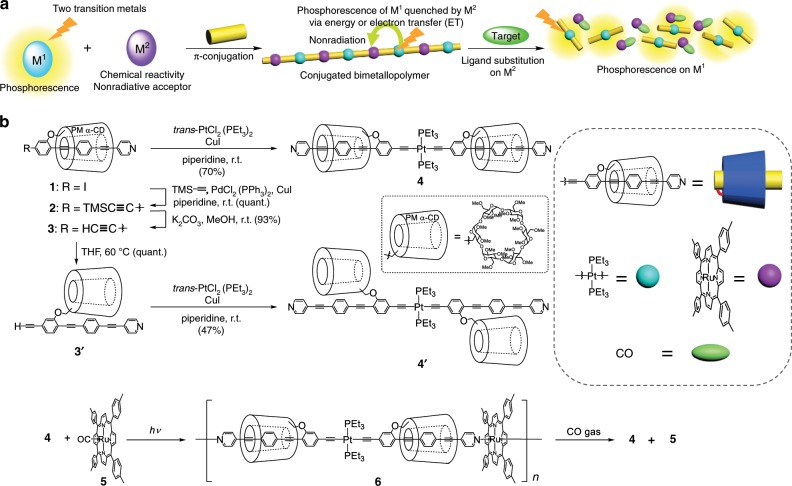
Synthesis and response of bimetallopolymer. **a** Illustrations of the phosphorescence sensing mechanism based on a conjugated bimetallopolymer with two transition metals. **b** The synthetic route to the insulated conjugated bimetallopolymer **6**.

Herein, a molecular design for the artificial dual self-controlling system, comprising self-activation and self-regulation, is proposed. We utilize the luminescence from the system as the output signal, specifically focusing on the phosphorescence of transition metal complexes. This is because the large stokes shift in their emission is largely unaffected by the excited light, which increases the signal-to-noise ratio^17–19^. To incorporate the dual responses into the phosphorescence output, a second transition metal was introduced. Accordingly, two transition metals, one capable of phosphorescence (M^1^), and the other behaving as a nonradiative acceptor with chemical reactivity (M^2^), were mutually connected with organic moieties to form a conjugated bimetallopolymer (Fig. 2a). In the polymer and oligomer, the excited state of M^1^ was thermally quenched by M^2^ via energy or electron transfer (ET)^20–23^. The ligand substitution on M^2^ with the target compounds resulted in depolymerization of the bimetallopolymer to phosphorescent monomers, M^1^, and target ligand bound-M^2^, thereby leading to monomer-based phosphorescence upon depolymerization (Fig. 1d). Here, M^1^ and M^2^ correspond to Pt(II)-acetylide and Ru(II) porphyrin-pyridyl moieties; that is, Pt(II)-acetylide functioned as the phosphorescent site^24^, while the Ru(II) porphyrin-pyridyl moieties behaved as acceptors with carbon monoxide (CO) reactivity^25^. As outlined in Fig. 1e, a hexacoordinated Ru complex allows switching of the target concentration dependence at the threshold concentration because the ligand substitution was proposed to follow a dissociation mechanism involving two elementary reactions, the reaction rate of one of which depended on the concentration of the target molecule^26^. Precisely, the biomimetic bimetallopolymer should respond to the concentration of the target gas, with the optical responses being separately activated and regulated at two threshold concentrations.

### Synthesis and optical properties of insulated conjugated bimetallopolymer 6

**Fig. 3:**
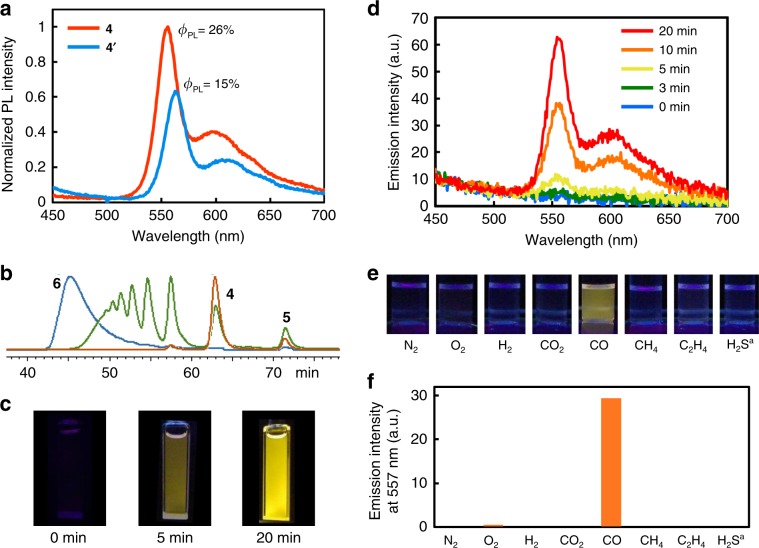
Optical analyses of Pt complexes and bimetallopolymer. **a** Emission spectra of Pt complexes **4** and **4ʹ** in deoxygenated toluene (concentration: 10^−5^ M). The spectra were corrected to the same number of photons absorbed at the excitation wavelength. **b** SEC profiles at various time points (blue: 0 min, green: 5 min, and orange: 20 min). **c** Photographic images upon excitation at 365 nm, and **d** emission spectra at a concentration of 10^−2^ mg/mL in deoxygenated toluene under excitation at 365 nm after depolymerization of the bimetallopolymer **6** with CO gas at various time points. **e** Photographic images and **f** emission intensities under deoxygenated conditions after depolymerization of the bimetallopolymer **6** with various gases under UV irradiation. ^a^1% (v/v) H_2_S was present in the N_2_ gas.

Figure 2b shows the synthetic route to the abovementioned conjugated bimetallopolymer containing an insulated structure, where the Pt(II) and Ru(II) complexes were polymerized with oligo(phenylene ethynylene) (OPE) backbones and insulated by permethylated α-cyclodextrins (PM α-CDs)^27,28^. According to our previous studies, the incorporation of rotaxane structures into conjugated metallopolymers enhanced the phosphorescence intensity^24^, thereby improving the output further. Precursor **1** was prepared in seven steps from 6-*O*-monotosyl PM α-CD, according to a previous report^27^. Compound **1** was alkynylated via a Sonogashira coupling reaction at ambient temperature to retain the insulated structure. Subsequent deprotection to remove the silyl group afforded the target insulated monomer **3**, where the insulated structure of **3** was kinetically stabilized by the terminal alkynyl group. The corresponding uninsulated monomer **3ʹ** was obtained by heating a solution of **3** in the hydrophobic solvent, tetrahydrofuran, which was unfavorable for the insulation^29^. A copper-catalyzed metalation reaction at room temperature between **3** and *trans*-[PtCl_2_(PEt_3_)_2_] formed a 2:1 insulated Pt-acetylide complex **4** (ref. ^30^), and the corresponding uninsulated complex **4ʹ** was also obtained under the same reaction condition from **3ʹ**. The ^1^H NMR spectrum of **4** displayed low-field shift for the insulated aryl groups and high-field shift for the pyridyl groups, as compared with the corresponding groups of **4ʹ** (Supplementary Fig. 1). The relative low-field shift occurred because of the supramolecular interaction between the PM α-CDs and the phenyl groups, while the high-field shift occurred because of the neighboring effect between the PM α-CDs and the pyridyl groups (Supplementary Fig. 2)^31^. In addition, the ^31^P NMR spectrum suggested that both the insulated and uninsulated Pt complexes adopted the *trans*-Pt-diacetylide conformation^32^. The clear vibration bands in deoxygenated toluene solutions were suggestive of π–π* phosphorescence, which was a characteristic luminescence of Pt-acetylide complexes (Fig. 3a). The lifetime of the excited species was ~1 μs, which was determined by transition absorption decay (Supplementary Figs. 6a and 7a)^20^. While the OPE conjugation in **4** was twisted to slightly shorten the effective conjugation length (**4**: 556 nm, **4ʹ**: 564 nm), insulation by the cyclic PM α-CDs protected the OPE conjugation from the thermal fluctuations^24,33^. The phosphorescence quantum yield of **4** (*Φ*_PL_ = 26%) in toluene was approximately twice that of **4ʹ** (*Φ*_PL_ = 15%). The unique enhancement in phosphorescence of the insulated structure could increase the optical intensity of the output.

A sequential complexation of the insulated monomer **3** with Pt(II) and Ru(II) formed the insulated conjugated bimetallopolymer **6**, as shown in Fig. 2b. The insulated Pt-acetylide complex **4** was co-polymerized with Ru(TTP)CO (**5**) (TTP: tetratolylporphyrin) to form a one-dimensional coordination polymer **6** (*M*_w_ = 1.3 × 10^5^, *M*_n_ = 4.5 × 10^4^), as confirmed by size exclusion chromatography (SEC) (The *M*_w_ and *M*_n_ of polymer **6** were obtained by polystylene calibration standard of reaction mixtures.). The carbonyl ligand on the Ru(II) complex was released as CO gas upon irradiation with a mercury lamp, affording *trans*-bispyridyl Ru(TTP)^25^. Similarly, **3** could be polymerized via sequential complexation with Ru(TTP)CO and then with *trans*-[PtCl_2_(PEt_3_)_2_] (see Supplementary Information). The sequence of complexation of the metals did not affect the formation of **6** because of the inherent stability of the metal–ligand bond. As a result, the supramolecular structure formed with the PM α-CDs was effectively retained even in the polymeric state. The metal–organic bonds on the bimetallopolymer remained intact even in a coordinating solvent (tetrahydrofuran). The optical behavior of the bimetallopolymer **6** differed significantly from that of Pt-acetylide monomer **4** (Supplementary Figs. 5–7). The excited-state species of **6** was quenched via a non-radiative processes on the porphyrin complex. The lifetime of the excited-state species of **6** dramatically decreased to 20 ns as compared with that of **4** (ref. ^34^), which was ~1 μs. Furthermore, the quantum yield of **6** decreased to 0.1% after polymerization, demonstrating the high ET efficiency (>99%) of the non-radiative pathway. Although several conjugated bimetallopolymers have been reported to date^35–41^, insulated bimetallopolymers bearing rotaxane structures have yet to be reported. Indeed, this is the first report on the synthesis of an insulated conjugated bimetallopolymer with rotaxane structures.

### Sigmoidal response by dual self-controlling system

To examine the optical response of the bimetallopolymer, a toluene solution of **6** was reacted with 1 atm CO gas at 100 °C for 20 min. The bimetallopolymer was stable under ambient conditions, while it was prone to reaction with CO at high temperatures (Supplementary Fig. 9). The strongly coordinating CO molecule generated the Ru(TTP)CO complex (**5**), subsequently releasing the Pt complex from it^42^. After the reaction, **6** was depolymerized to **4** and **5**, as indicated in the SEC profiles (Fig. 3b). While the emission spectrum of **6** indicated that the reaction between **4** and the Ru complex completely quenched the phosphorescence as compared with that of **4**, the resultant solution displayed yellow phosphorescence that was identical to the phosphorescence of **4** when excited at 365 nm (Fig. 3c and d). This afforded a turn-on optochemical response triggered by CO gas. Additionally, UV irradiation of the depolymerized solution with a mercury lamp for 6 h resulted in the repolymerization, as confirmed by SEC (*M*_w_ = 6.9 × 10^4^, *M*_n_ = 2.5 × 10^4^) (Supplementary Fig. 10). The decline in the polymerization degree could be attributed to the slight degradation of the monomer components. Moreover, the bimetallopolymer solution remained intact upon exposure to 1 atm of various other gases at 100 °C for 10 min, including oxidative gas (O_2_) and reductive gases (H_2_, C_2_H_4_), demonstrating its chemospecific reactivity toward CO gas (Fig. 3e, f, and Supplementary Fig. 11). It was noteworthy that the bimetallopolymer responded to 1% CO gas in ambient air to display phosphorescence, while it was intact to ambient air in the absence of CO gas (Supplementary Figs. 12 and 13), indicating that the responsiveness of bimetallopolymer to CO gas was not affected, even under mixed gas conditions. These results supported that the CO molecule selectively cleaved the Ru-pyridyl bonds on **6** without affecting the polymer components. Thus, Ru and Pt took part in imparting chemical reactivity and phosphorescence, respectively, in the optochemical response to CO gas.

**Fig. 4:**
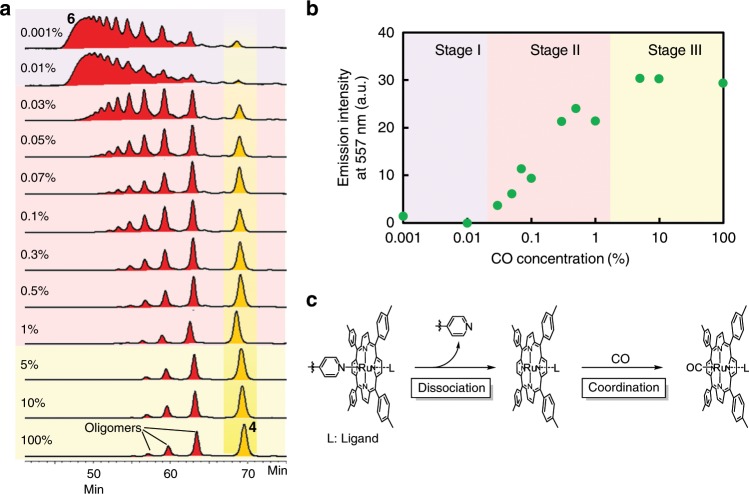
Optical output with the dual self-controlling system. **a** SEC profiles (UV detector, 380 nm) and **b** concentration dependence of the emission intensities at 557 nm (deoxygenated condition, excitation at 365 nm) after reaction at 100 °C for 10 min with various concentrations of CO gas. **c** Ligand substitution reaction of the Ru(TTP)(L)Py complex with CO (Py: pyridine, L: ligand).

The concentration dependence of the depolymerization of **6** on CO gas was examined. The phosphorescence intensities were measured after heating a solution of **6** in toluene at 100 °C for 10 min under various concentrations of CO gas (in N_2_), ranging from 0.001 to 100%. At low concentrations (< 0.01%) (0.001% CO gas in N_2_ approximately corresponded to 4 equivalents to the Ru complexes in the solution of bimetallopolymer), the emission was independent of the concentration, although depolymerization still occurred to some extent, as confirmed by SEC analyses (Fig. 4a); however, no emission was obtained (Fig. 4b). In this region (Stage I), the slight depolymerization upon a random cleavage of the Ru complexes of the polymer only afforded non-radiative bimetallic polymers and oligomers, and not the phosphorescent Pt monomer **4**. This was attributed to the effective ET among the adjacent multiple metals, which is a unique feature of bimetallopolymers (Supplementary Figs. 5–7). The independency afforded the first threshold for the gas concentration and prevented the needless responses to trivial inputs. In the next region (Stage II), the luminescent intensity increased with the CO concentration (0.03–1%), which was an autonomous response above the threshold (self-activation). On the other hand, at high concentration (5–100%, Stage III), the emission intensity was again independent of the CO concentration, affording the second threshold of the output for the gas concentration (Fig. 4b). The second threshold provided a biomimetic auto-modulation for excess inputs (self-regulation). In the following region, the progress of the reaction was constant when the CO gas concentration was increased from 5 to 10% and again to 100%; the same degree of depolymerization was observed by SEC, although the reactions did not reach completion (Fig. 4a). The incomplete depolymerization was also indicated by the lower emission intensity (~30) as compared with that of the fully converted product (~60) with the same polymer concentration (Fig. 3d). The depolymerizing ligand exchange reaction from Ru(TTP)(L)Py (Py: pyridine, L: ligand) to Ru(TTP)(L)CO proceeded via a two-step dissociation mechanism on the hexacoordinated Ru center (Fig. 4c)^26^. At concentrations below 1%, the rate-determining step of depolymerization was the CO coordination step. The extent of progress of the reaction gradually increased with the CO concentration (0.03–1%). In contrast, in a CO concentration range of 5–100%, the CO coordination step was sufficiently rapid to not govern the total reaction rate, and hence, the rate-determining step of depolymerization was dissociation of the pyridyl groups; the extent of progress of the reaction was independent of the CO concentration.

### Tunable thresholds for dual self-controlling system

**Fig. 5:**
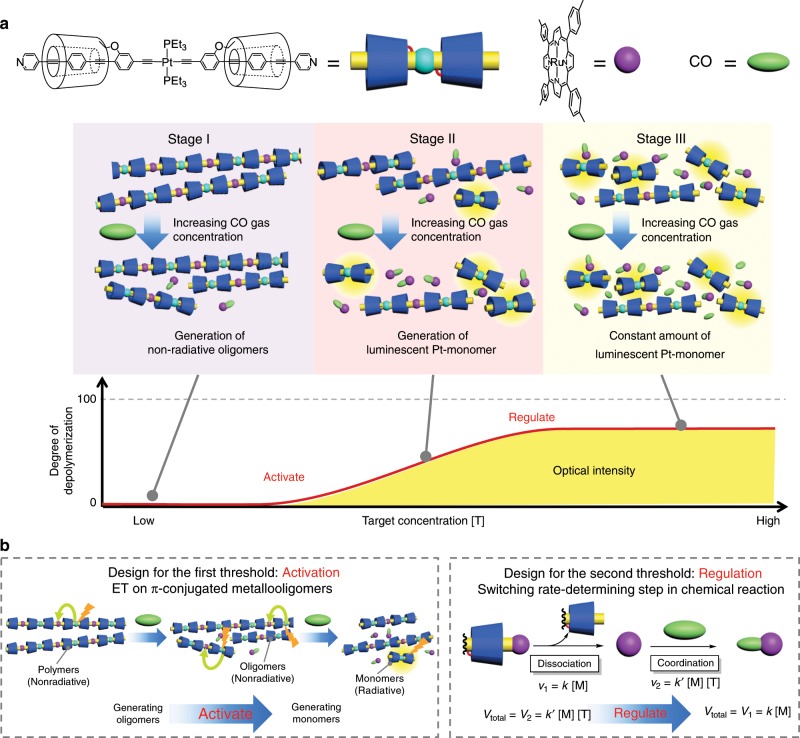
Summary of the depolymerization steps with two thresholds. **a** Change in the optical intensity with increasing target concentrations. **b** The design principle for self-activation and self-regulation ([M]: concentration of sensing metal, [T]: target concentration, *k* and *k*′ reaction rate constants).

**Fig. 6:**
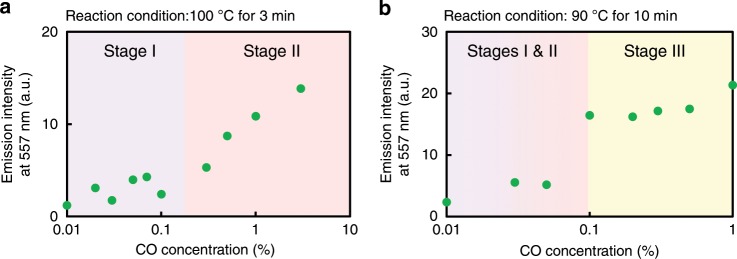
Tunable thresholds in dual self-controlling system. Concentration dependence of the emission intensities at 557 nm (deoxygenated condition, excitation at 365 nm) after reactions with various concentrations of CO gas; **a** at 100 °C for 3 min and **b** at 90 °C for 10 min.

Owing to the two threshold concentrations, the bimetallopolymer displayed a dual self-controlling emission response to the CO gas concentration (Fig. 5a, b). Furthermore, the thresholds for modulation could be controlled by altering the reaction conditions. For example, the first threshold for activation was controlled by changing the reaction time. In this case, the phosphorescent intensities were measured after heating a solution of the bimetallopolymer in toluene at 100 °C for 3 min under various concentrations of CO gas, ranging from 0.01 to 3%. Above a concentration of 0.1%, the emission intensities increased with the CO concentration (e.g. Stage II), while below 0.1%, the emission intensities were independent of the CO concentration (Stage I) (Fig. 6a). The second threshold for regulation was controlled by varying the reaction temperature. More specifically, after heating the solution at 90 °C for 10 min under various concentrations of CO gas, ranging from 0.03 to 1%, a decrease in the temperature reduced the rate of the Ru complex dissociation reaction. Above 0.1% (Stage III), the emission intensities were constant (Fig. 6b), which demonstrates switching of the rate-determining step at the threshold. The SEC profiles also supported the trends (Supplementary Fig. 16). Overall, these results confirm that the first and second threshold concentrations could be shifted by altering the reaction time and temperature, respectively. Such separate threshold values for low and high concentrations are common in biological regulatory systems. For example, neurotransmitters, ion channels, and immune systems adjust their responses by promoting and regulating their outputs to external stimuli as positive aerostatic effects or competitive inhibitions, affording non-linear behaviors. The bimetallopolymer is one such biomimetic material in a single polymer, which was prepared by the precise engineering of artificial molecular systems: the first threshold promoted the phosphorescence output, while the second regulated it, thereby adjusting and prohibiting an excess response.

## Discussion

In summary, the insulated conjugated bimetallopolymer behaved as a self-activating and self-regulating biomimetic material responding to CO gas by exhibiting unusual, distinct signal changes at two threshold concentrations. The self-activation was driven by ET after excitation of the π-conjugated bimetallopolymer and oligomers, affording the first threshold via monomer-induced emission. In contrast, the self-regulation was driven by dissociative ligand substitution on the reactive metal complex, affording the second threshold via a switch in the rate-determining step. We expect that the incorporation of other metal complexes in this strategy could enable control of the response temperature, the target gases, and the concentration range, thereby provide further diversity on terms of the responses and the operating environment for such biomimetic materials. The strategies for incorporating multiple self-modulating systems into irreversible reactions would provide novel molecular designs for controlling the detection, manufacturing, and transferring of chemicals. Such biomimetic controlling would also lead to unprecedented systems in diverse areas of materials chemistry, e.g., autonomous modulation, to automatically tune the outputs depending on the external environment without any requirement for human control.

## Methods

### Synthesis of 4

Under argon, **3** (133 mg, 88.0 μmol), *trans*-PtCl_2_(PEt_3_)_2_ (21.5 mg, 42.8 μmol), and CuI (1.9 mg, 10 μmol) were dissolved in degassed piperidine (5 mL). The reaction mixture was stirred at room temperature for 2 h. The mixture was quenched with aqueous NH_4_Cl and diluted with CHCl_3_. The organic layer was separated and dried over MgSO_4_, and then filtered. The solvent was removed by evaporation, and the residue was purified by GPC with CHCl_3_ as the eluent to yield **4** as a yellow solid (102 mg, 70%). MALDI TOF-MS: (*m*/*z*) 3475.4682 ([M + Na^+^]^+^, C_164_H_238_N_2_O_60_P_2_PtNa, calcd. 3475.4661). ^1^H NMR (500 MHz, CDCl_3_, r.t.) *δ* 8.64 (br, 4H, PyH), 8.08 (d, *J* = 8.2 Hz, 4H, ArH), 7.63 (d, *J* = 7.9 Hz, 4H, ArH), 7.34 (br, 4H, PyH), 7.30 (d, *J* = 7.9 Hz, 2H, ArH), 7.02 (d, *J* = 8.2 Hz, 2H, ArH), 7.00 (s, 2H, ArH), 5.11–2.85 (m, 186H, CD-H, OCH_3_), 2.17–2.15 (m, 12H, PCH_2_), 1.26–1.19 (m, 18H, PCH_2_CH_3_). ^13^C NMR (126 MHz, CDCl_3_, r.t.): *δ* 161.81, 149.83, 132.90, 132.43, 131.53, 131.13, 131.01, 125.93, 125.60 (peaks overlapped), 124.08, 123.61, 122.32, 112.95, 109.51, 100.80, 100.45, 100.21, 100.09, 100.01, 98.13, 93.71, 93.01, 90.03, 88.25, 83.83, 82.78, 82.60 (peaks overlapped), 82.55, 82.40, 82.21, 82.18 (peaks overlapped), 82.14, 82.02, 81.70, 81.48, 81.29 (peaks overlapped), 81.22, 81.18, 81.07, 76.22, 72.25, 71.94, 71.72, 71.55 (peaks overlapped), 71.28 (peaks overlapped), 71.17, 71.08, 70.72, 70.15, 61.94, 61.82 (peaks overlapped), 61.74, 61.71, 61.58, 59.05 (peaks overlapped), 58.96, 58.67, 58.64, 58.40, 58.05, 57.89, 57.76, 57.69, 57.56, 16.45 (t, ^1^*J*_C-P_ = 35.2 Hz), 8.31. ^31^P{^1^H} NMR (160 MHz, CDCl_3_, r.t.): *δ* 11.17 (s + d, ^1^*J*_P-Pt_ = 2353 Hz).

### Synthesis of 4′

Under argon, **3** (80 mg, 53 μmol) was dissolved in degassed tetrahydrofuran (5 mL). The solution was stirred at 60 °C overnight. The solvent was removed by evaporation to yield **3ʹ** as a yellow solid (80 mg, quant.) without further purification. Under argon, **3ʹ** (50 mg, 33 μmol) and *trans*-PtCl_2_(PEt_3_)_2_ (7.9 mg, 16 μmol) and CuI (0.6 mg, 3 μmol) were dissolved in degassed piperidine (3 mL). The reaction mixture was stirred at room temperature for 4 h. The mixture was quenched with aqueous NH_4_Cl and diluted with CHCl_3_. The organic layer was separated and dried over MgSO_4_, and then filtered. The solvent was removed by evaporation, and the residue was purified by GPC with CHCl_3_ as the eluent to yield **4ʹ** as a yellow solid (27 mg, 47%). MALDI TOF-MS: (*m*/*z*) 3475.4659 ([M + Na^+^]^+^, C_164_H_238_N_2_O_60_P_2_PtNa, calcd. 3475.4661). ^1^H NMR (500 MHz, CDCl_3_, r.t.): *δ* 8.77 (br, 4H, PyH), 7.63 (d, *J* = 8.2 Hz, 4H, ArH), 7.50 (d, *J* = 8.2 Hz, 4H, ArH), 7.47 (br, 4H, PyH), 7.29 (d, *J* = 7.9 Hz, 2H, ArH), 6.85 (d, *J* = 7.9 Hz, 2H, ArH), 6.76 (s, 2H, ArH), 5.22–3.02 (m, 186H, CD-H, OCH_3_), 2.13–2.12 (m, 12H, PCH_2_), 1.23–1.17 (m, 18H, PCH_2_CH_3_). ^13^C NMR (126 MHz, CDCl_3_, r.t.): *δ* 159.11, 149.70, 132.71, 131.73, 131.60, 131.23, 130.45, 124.76, 124.04, 121.22, 113.92, 111.35, 111.23, 110.32, 109.65, 100.68, 100.24, 100.17, 100.14, 100.10, 99.42, 93.82, 93.55, 89.62, 88.22, 82.69, 82.59, 82.52 (peaks overlapped), 82.49, 82.33, 82.30, 82.24, 82.20, 82.18, 82.08, 81.58, 81.23 (peaks overlapped), 81.19 (peaks overlapped), 81.11, 71.92, 71.78, 71.58 (peaks overlapped), 71.55, 71.47, 71.30 (peaks overlapped), 71.18 (peaks overlapped), 70.96, 67.51, 61.86, 61.83 (peaks overlapped), 61.81, 61.79, 61.76, 59.15 (peaks overlapped), 59.07, 59.03 (peaks overlapped), 58.37, 57.93, 57.90 (peaks overlapped), 57.83, 57.35, 16.38, (t, ^1^*J*_C-P_ = 33.9 Hz), 8.37. ^31^P NMR (202 MHz, CDCl_3_, r.t.): *δ* 11.56 (s + d, ^1^*J*_P-Pt_ = 2365 Hz).

### Synthesis of 6

Compound **4** (20 mg, 5.8 μmol) and [Ru(TTP)CO] (**5**: 4.9 mg, 5.8 μmol) were dissolved in toluene (20 mL). The reaction mixture was irradiated using a high-pressure mercury lamp for 8 h under argon bubbling and stirring at room temperature. As the reaction was proceeded, the solution color was changed from red to purple. The solvent was removed by evaporation to yield **6** as a purple solid. The bimetallopolymer was used for the following experiments without further purifications (0.2 mg: *M*_w_ = 1.3 × 10^5^, *M*_n_ = 4.5 × 10^4^). ^1^H NMR (500 MHz, CDCl_3_, r.t.): *δ* 8.16 (br, 8H, β-H), 7.89 (br,12H, tol-H, ArH), 7.44 (br, 10H, tol-H, ArH), 7.25–7.10 (br, 4H, ArH), 6.93 (br, 4H, ArH), 5.29 (br, 4H, PyH), 5.04–2.77 (m, 186H, CD-H), 2.62 (br s, 12H, tol-CH_3_), 2.25 (br, 4H, PyH), 2.10–2.08 (br m, 12H, PCH_2_), 1.17–1.15 (br m, 18H, PCH_2_C*H*_3_). ^31^P NMR (202 MHz, CDCl_3_, r.t.): *δ* 11.20 (s + d, ^1^*J*_P-Pt_ = 2359 Hz).

### General procedure of reaction of bimetallopolymer 6 with CO gas

Bimetallopolymer **6** (0.2 mg) was added into a 20-mL two-necked round-bottom flask (40 mL total volume). The flask was filled with CO gas and then was added toluene (1 mL). The mixture was stirred at 100 °C for 20 min. As the reaction was proceeded, the solution color changed from purple to red. The reaction was monitored by analytical SEC chromatogram to confirm the decomplexation of bimetallopolymer.

### Recycling procedure

Bimetallopolymer **6** (0.2 mg: *M*_w_ = 1.3 × 10^5^, *M*_n_ = 4.5 × 10^4^) was added into a 20-mL two-necked round-bottom flask (40 mL total volume). The flask was filled with CO gas and then was added toluene (1 mL). The mixture was stirred at 100 °C for 20 min. The reaction was monitored by analytical SEC chromatogram to confirm the decomplexation of bimetallopolymer. After the depolymerization, the resultant mixture without any purification was irradiated using a mercury lamp for 1.5 h under N_2_ bubbling and stirring at room temperature to change the solution color from red to purple. The reaction was monitored by analysis SEC chromatogram to confirm the recomplexation to form bimetallopolymer (*M*_w_ = 6.9 × 10^4^, *M*_n_ = 2.5 × 10^4^).

### Procedure of reactions with various gases

Bimetallopolymer **6** (0.2 mg) was added into a 20-mL two-necked round-bottom flask (40 mL total volume). The flask was filled with target gas (N_2_, O_2_, H_2_, CO_2_, CO, CH_4_, C_2_H_4_, and 1% v/v H_2_S in N_2_ gases) and then was added toluene (0.5 mL). The solution was stirred at 100 °C for 10 min. The flask was immediately cooled to room temperature to prohibit the reaction.

### Procedure of analyses for concentration dependence

Bimetallopolymer **6** (0.2 mg) was added into a 20-mL two-necked round-bottom flask (40 mL total volume). The flask was filled with N_2_ gas and then was added toluene (0.5 mL). CO (>99.95%) gas was added into the flask to provide the desired concentrations. The solutions were stirred at 100 °C for 10 min. The flask was immediately cooled to room temperature to prohibit the CO gas reaction. The emission spectra were measured under nitrogen atmosphere, after excluding the reaction gas.

## Supplementary information

Supporting information PDF file

## Data Availability

All other data that support the findings of this study are available within the article and its Supplementary Information, or from the corresponding author upon reasonable request.
