# Supplementary material for: Insulated conjugated bimetallopolymer with sigmoidal response by dual self-controlling system as a biomimetic material
Source: Nat Commun. 2020 Jan 21;11:408. doi: 10.1038/s41467-019-14271-2 (PMC6972936; doi:10.1038/s41467-019-14271-2)
Supplement: Supplementary file 1 — Supporting information PDF file [file 41467_2019_14271_MOESM1_ESM.pdf]

Supplementary Information

Insulated conjugated bimetallopolymer with sigmoidal response by dual self-controlling system as a biomimetic material

Masai et al.

Supplementary Note 1
General Remarks

Material: Unless otherwise stated, commercially available chemicals were used as received. Piperidine was degassed through ether argon or nitrogen bubbling, before use. Toluene and THF were purchased from Kanto Chemical and further purified by passage through activated alumina under positive argon or nitrogen pressure as described by Grubbs et al. [1] The monomer precursor **1**[2] and Ru(TTP)CO (**5**)[3] were prepared according to our previously reported procedures. The Ru complex **5** was estimated to bond EtOH on Ru as a ligand because it was recrystallized with CH_2Cl_2-EtOH (49:1)/hexane solvent system, before use.

NMR Spectroscopy: ^{1}H NMR (500 MHz), ^{13}C {^{1}H } NMR (126 MHz), and ^{31}P{^{1}H} NMR (202 MHz) were measured with a Bruker AVANCE-500 spectrometer. The ^{1}H NMR chemical shifts are reported relative to tetramethylsilane (TMS, 0.00 ppm) or residual protonated solvents (7.26 ppm) in $CDCl_3$ or (2.08 ppm) in toluene-d_8. The ^{13}C NMR chemical shifts are reported relative to $^{13}CDCl_3$ (77.0 ppm). The ^{31}P{^{1}H} NMR spectra were recorded using 85% H_3PO_4 as an external standard.

Mass Spectroscopy (MS): Matrix assisted laser desorption/ionization (MALDI) mass spectra were obtained with α-cyano-4-hydroxycinnamic acid or *trans*-2-[3-(4-*tert*-butylphenyl)-2-methyl-2-propenylidene]malononitrile as matrixes and Na^+ as a cationization reagent on Thermo Fisher Scientific LTQ orbitrap XL.

Preparative Recycling Gel Permeation Chromatography (GPC): Preparative recycling GPC was performed with a SHIMADZU LC-20AP System equipped with a Shodex K-4003L, K-4002.5L, K-4002L, or K-4001L column, a SHIMADZU SPD-20A, and a SHIMADZU RID-10A using $CHCl_3$ as the eluent at a flow rate of 14 mL min^{-1}.

Analytical Size-exclusion Chromatography (SEC): Analytical SEC was performed with a GL-Science GL-7400 HPLC System equipped with Shodex KF-801, -802, -802.5, -803, -804 columns, a GL-7410 HPLC pump, a GL-7400 UV detector, and a GL-7454 RI detector using THF as the eluent at a flow rate of 0.6 mL min^{-1}.

Thermogravimetric analysis: Thermogravimetric analyses were conducted with a Rigaku Thermo plus TG8120 system.

Absorption Spectra: The solution samples were prepared in concentrations of 10^{-2} mg/mL in toluene except to the investigation for the concentration dependence. Ultraviolet-visible absorption spectra

were measured with a SHIMADZU UV-2600 model.

Emission Spectra/Quantum Yield: The solution samples were prepared in concentrations of 10^{-2} mg/mL in toluene and then degassed through N_2 bubbling. Fluorescent spectra and absolute quantum yields were obtained using a Hamamatsu C11347 instrument with a calibrated integrating sphere system. The slit width for irradiation was 1.2 mm and the exposure time was automatically calibrated.
Transient Absorption Spectra (TAS): The solution samples were prepared in concentrations of 10^{-2} mg/mL in toluene for measuring their spectra.
Nanosecond TAS measurements were performed by using a home-built nanosecond TAS system with a N_2 laser (LTB Lasertechnik Berlin GmbH, MNL 202-C) pumped dye laser (LTB Lasertechnik Berlin GmbH, ATM200, 700 ps pulse duration) as an excitation source. Transient absorption signal was probed by a Xe lamp (Photon Technology International) light through two monochromators (Acton, Princeton Instruments), and detected by a Si based nanosecond detection system (Unisoku Co., Ltd., TSP-2000SN, time resolution: 1.2 ns (FWHM), monitoring wavelengths: 400~1,100 nm) with a fast oscilloscope (Tektronix, TDS 3052C, Digital Phosphor Oscilloscope 500 MHz 5 GS/s) at 2 Hz excitation repetition rate. Transient data were collected with 337 nm excitation with excitation intensity of 70 μJ cm^{-2} at 22 °C.
Microsecond TAS measurements were performed with a home-built TAS system with a N_2 laser (OBB, OL-4300) pumped dye laser (OBB, OL-401, 800 ps pulse duration) as an excitation source. The data was obtained with a photodiode-based detection system (Costronics Electronics) through a monochromator (JASCO, M10), and the signal was collected through a TDS-2022 Tektronix oscilloscope. Measurements were conducted with a pulse excitation energy density of 70 μJ/cm^2 with a repetition rate of 2 Hz at 22 °C. The instrument response time was ~700 ns. Transient spectra were corrected for the spectral response of a grating in a monochromator and a detector. No change in the steady-state absorption spectra before and after the transient experiments was observed, suggesting that the samples were stable during the experiments.

Gas Preparation: CO_2 (99.9% purity), O_2 (99.9% purity), H_2 (99.99% purity), CH_4 (99.9% purity), and C_2H_4 (99.5% purity) gases were supplied by portable gas canisters which were purchased from GL Science. CO (>99.95% purity) gas cylinder was purchased from Taiyo Nippon Sanso. N_2 was prepared via vaporization of liquid N_2 (99.999% purity) purchased from ATOX. H_2S gas was prepared via a laboratory method ($FeS + H_2SO_4$ (dil.) $\rightarrow H_2S + FeSO_4$), and then was passed through $CaCl_2$ powder to trap out H_2O contaminants.

Supplementary Methods

Synthesis of 2

Under argon, **1** (189 mg, 117 μmol), $PdCl_2(PPh_3)_2$ (4.2 mg, 5.9 μmol) and CuI (1.1 mg, 5.9 μmol) were added into degassed piperidine (2.5 mL). Into the solution, trimethylsilylacetylene (80.8 μL, 569 μmol) was added, and then the reaction mixture was stirred at room temperature for 18 h. The mixture was quenched with aqueous NH_4Cl and diluted with $CHCl_3$. The organic layer was separated and dried over $MgSO_4$, and then filtered. The solvent was removed by evaporation, and the residue was purified by GPC with $CHCl_3$ as the eluent to yield **1** as a yellow solid (192 mg, quant.).

MALDI TOF-MS: (*m*/*z*) 1606.7004 ($[M+Na^+]^+$, $C_{79}H_{113}NO_{30}SiNa$, calcd. 1606.7009). ^1H NMR (500 MHz, $CDCl_3$, r.t.): δ 8.64 (br, 2H, PyH), 8.10 (d, J = 8.2 Hz, 2H, ArH), 7.64 (t, J = 7.9 Hz, 2H, ArH), 7.45 (d, J = 7.9 Hz, 1H, ArH), 7.34 (br, 2H, PyH), 7.24 (s, 1H, ArH), 7.22 (d, J = 7.9 Hz, 1H, ArH), 5.10-2.86 (m, 93H, CD-H, OCH_3), 0.27 (s, 9H, $SiCH_3$). ^{13}C NMR (126 MHz, $CDCl_3$, r.t.): δ 161.75, 149.98, 133.16, 132.80, 131.70, 131.05, 126.90, 125.54, 125.39, 123.21, 122.93, 116.97, 103.64, 100.89, 100.66, 100.35, 100.28, 100.08, 98.25, 97.64, 94.96, 92.96, 89.10, 88.60, 83.92, 83.06, 82.91, 82.68 (peaks overlapped), 82.54, 82.48, 82.30, 82.26, 82.23, 82.15, 81.77, 81.60, 81.41 (peaks overlapped), 81.35 (peaks overlapped), 81.22, 76.65, 72.50, 72.09, 71.73, 71.68, 71.50, 71.44, 71.33 (peaks overlapped), 70.77, 70.35, 62.08, 61.96 (peaks overlapped), 61.85 (peaks overlapped), 61.72, 59.24, 59.20, 59.08, 58.92, 58.89, 58.54, 58.19, 58.02, 57.86, 57.83, 57.70, -0.16.

Synthesis of 3

Under argon, **2** (150 mg, 94.7 μmol) and K_2CO_3 (39.1 mg, 283 μmol) were dissolved in MeOH (20 mL). The reaction mixture was stirred at room temperature for 1 h. The mixture was quenched with H_2O, and diluted with $CHCl_3$ and washed with brine. The organic layer was separated and dried over $MgSO_4$. The solvent was removed by evaporation and the residue was purified by GPC with $CHCl_3$ as the eluent to yield **3** as a yellow solid (133 mg, 93%). MALDI TOF-MS: (*m*/*z*) 1534.6602 ($[M+Na^+]^+$, $C_{76}H_{105}NO_{30}Na$, calcd. 1534.6614). ^1H NMR (500 MHz, $CDCl_3$, r.t.): δ 8.67 (br, 2H, PyH), 8.10 (d, J = 8.2 Hz, 2H, ArH), 7.64 (d, J = 8.2 Hz, 2H, ArH), 7.49 (d, J = 8.5 Hz, 1H, ArH), 7.36 (br, 2H, PyH), 7.29 (s, 1H, ArH), 7.28 (s, 1H, ArH), 5.09-2.90 (m, 94H, CD-H, OCH_3, CCH). ^{13}C NMR (126 MHz, $CDCl_3$, r.t.): δ 161.63, 149.78, 133.29, 132.66, 131.81, 131.74, 131.56, 129.59, 128.68, 126.96, 125.51, 124.13, 122.99, 122.87, 117.34, 100.75, 100.51, 100.21, 100.15, 99.95, 98.13, 94.94, 92.77, 88.79, 88.51, 83.79, 82.97, 82.80, 82.53, 82.40, 82.34, 82.19, 82.16, 82.12, 82.02, 81.69, 81.47, 81.27 (peaks overlapped), 81.22, 81.20, 81.08, 79.86, 76.54, 72.32, 71.96, 71.89, 71.60, 71.54, 71.36 (peaks overlapped), 71.24, 71.17, 70.66, 70.31, 61.93, 61.81 (peaks overlapped), 61.71 (peaks overlapped), 61.58, 59.07, 59.03, 58.93, 58.74, 58.68, 58.41, 58.06, 57.88, 57.73, 57.69, 57.57.

Another synthetic route to bimetallopolymer **6**

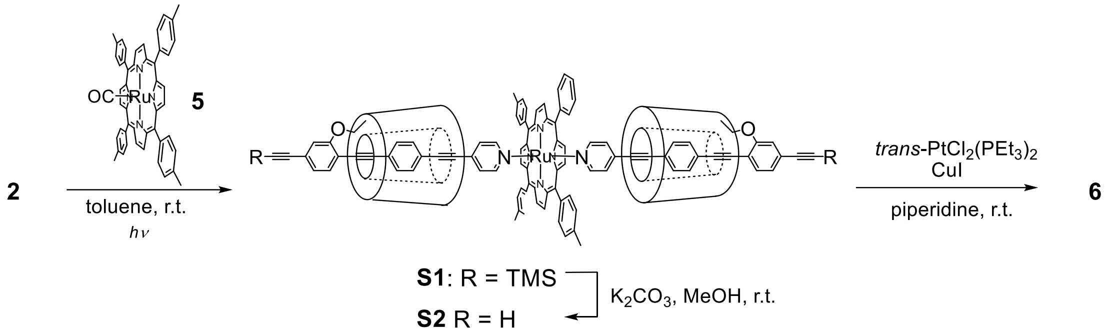

Synthesis of **S2**

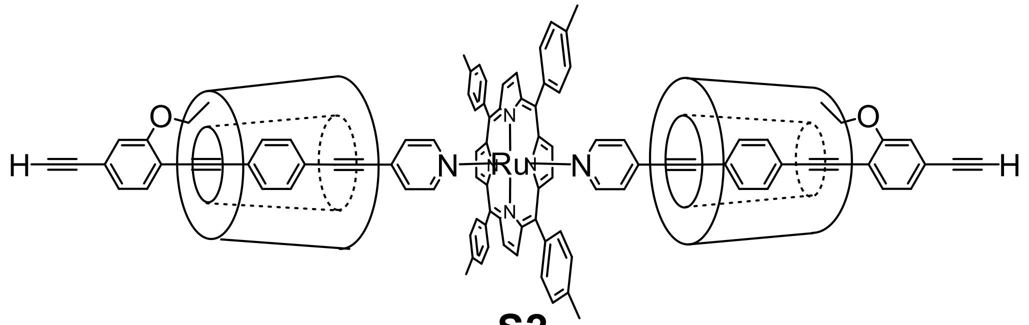

S2

2 (100 mg, 63.1 μmol) and [Ru(TPP)CO] (24.0 mg, 28.0 μmol) were dissolved in toluene (75 ml). The reaction mixture was irradiated using a high-pressure mercury lamp for 22 h under argon bubbling and stirring at room temperature. As the reaction was proceeded, the solution color was changed from red to purple. The solvent was removed by evaporation and the residue was purified by GPC with $CHCl_3$ as the eluent to yield **S1** as a purple solid (97 mg, 78%).

Under an argon, **S1 (**85.0 mg, 22.0 μmol) and K_2CO_3 (11.8 mg, 85.5 μmol) was dissolved in MeOH (3 mL) and THF (15 mL). The reaction mixture was stirred at room temperature for 3.5 h. The mixture was quenched with H_2O, and diluted with $CHCl_3$ and washed with brine. The organic layer was separated and dried over $MgSO_4$. The solvent was removed by evaporation and the residue was purified by GPC with $CHCl_3$ as the eluent to yield **S2** as a purple solid (63 mg, 77%).

MALDI HR-MS: (*m*/*z*) 3793.5461 ($[M]^{•+}$, $C_{200}H_{246}N_6O_{60}Ru$, calcd. 3793.5467). ^{1}H NMR (500 MHz, $CDCl_3$, r.t.): δ 8.10 (br, 8H, β-H), 7.90 (d, J = 7.6 Hz, 8H, tol-H), 7.85 (d, J = 8.2 Hz, 4H, ArH), 7.44 (d, J = 7.6 Hz, 8H, tol-H), 7.40 (d, J = 8.5 Hz, 2H, ArH), 7.21-7.20 (m, 4H, ArH), 7.15 (d, J = 8.2 Hz, 4H, ArH), 5.20 (br, 4H, PyH), 4.98-2.77 (m, 188H, CD-H, OCH_3, CCH), 2.64 (s, 12H, CH_3), 2.29 (br, 4H, PyH). ^{13}C NMR (126 MHz, $CDCl_3$, r.t.): δ 161.53, 149.93, 143.84, 139.70, 136.42, 136.38, 133.86, 133.18, 132.37, 132.18, 131.41, 131.04, 129.03, 128.23, 127.14, 126.89, 125.44, 125.30, 123.99, 122.59, 122.39, 122.14, 121.68, 117.28, 100.70, 100.45, 100.15, 100.04, 99.88, 98.06, 94.81, 93.38, 88.64, 87.34, 83.67, 82.86, 82.67, 82.39, 82.31, 82.26, 82.04 (peaks overlapped), 81.98, 81.90, 81.53, 81.36, 81.19, 81.13, 81.09, 81.06, 80.94, 79.75, 76.36, 72.21, 71.83 (peaks overlapped), 71.47, 71.41,

71.24 (peaks overlapped), 71.15, 71.07, 70.56, 70.21, 61.77, 61.69 (peaks overlapped), 61.62, 61.55, 61.44, 58.99, 58.96, 58.86, 58.66, 58.60, 58.29, 57.96, 57.80, 57.63, 57.61, 57.47, 21.46.

Another synthetic method of **6**

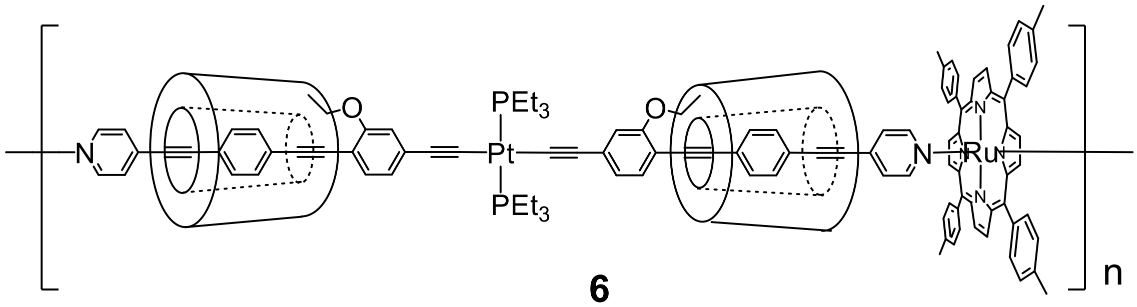

6

Under argon, **S2** (12 mg, 3.0 μmol) and *trans*-$PtCl_2$ $(PEt_3)_2$ (1.5 mg, 3.0 μmol) and CuI (0.06 mg, 0.3 μmol) were dissolved in degassed piperidine (2 mL). The reaction mixture was stirred at room temperature for 5 h. The mixture was quenched with aqueous NH_4Cl and diluted with $CHCl_3$. The organic layer was separated and dried over $MgSO_4$, and then filtered. The solvent was removed by evaporation, and the residue ($M_w = 5.5\times10^4$, $M_n = 2.4\times10^4$) was purified by GPC with $CHCl_3$ as the eluent to yield **6** as a purple solid (10 mg, 78%). Spectra were in good agreement with that mentioned above.

Supplementary Note 2

Chemical shift in rotaxane structure

Insulated (**4**) and uninsulated (**4′**) Pt complexes indicated their characteristic chemical shifts in aromatic region of ^{1}H NMR spectra according to their supramolecular structures. The ^{1}H NMR spectrum of **4** displayed low-field shift in the insulated aryl groups and high-field shift in the pyridyl groups as compared to that of **4′** (Supplementary Fig. 1). The shifts attributed to supramolecular interaction between PM α-CDs and phenyl groups and to neighboring effect between the lops of PM α-CDs and pyridyl groups as shown in an optimized structure of **4** with ONIOM calculations (Supplementary Fig. 2).

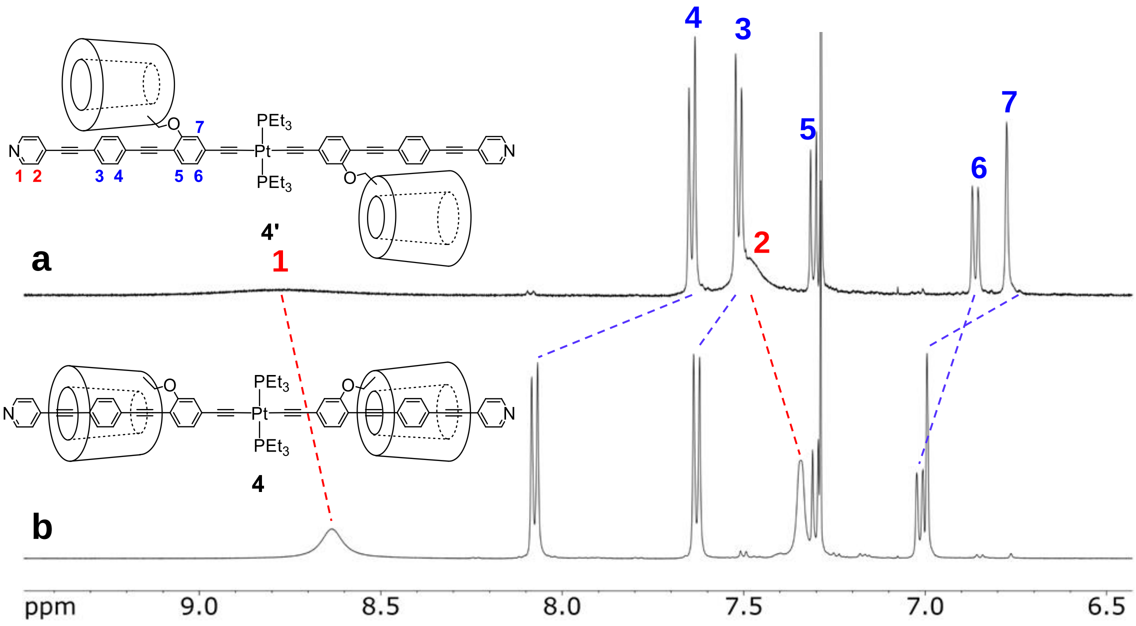

Supplementary Figure 1 ^{1}H NMR spectra (500 MHz, $CDCl_3$, r.t.) in aromatic region of Pt complexes; **a** uninsulated complex **4′** and **b** insulated complex **4**.

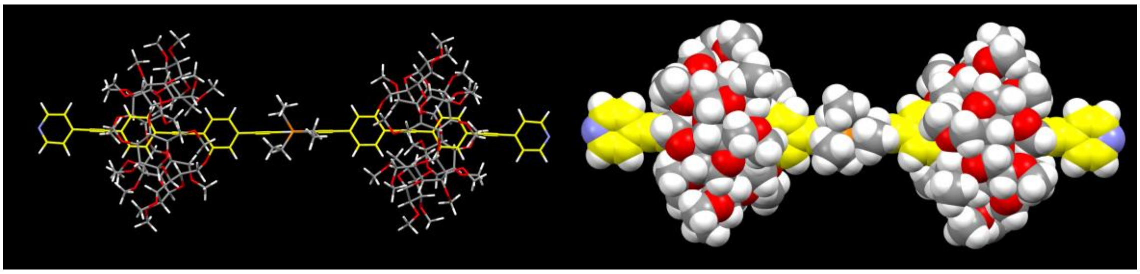

Supplementary Figure 2 Stick model and space filling model of a calculated structure of **4** using ONIOM (B3LYP/LanL2DZ-PM6).

Supplementary Note 3

Thermal stability of rotaxane structure

Different chemical shifts were shown between insulated monomer **4** and uninsulated monomer **4′** in ^{1}H NMR spectra, especially in the aromatic region; aromatic protons showed upfield shifts due to insulation of PM α-CDs. The solution of **4** in toluene-d_8 (hydrophobic solvent) was heated under 100 °C for 20 min and for 1 h. The aromatic protons and others in ^{1}H NMR spectrum were retained after reaction, and no peaks corresponding to uninsulated monomer **4′** were observed in the aromatic region (Supplementary Fig. 3). These results indicated that the rotaxane structure of monomer **4** possessed a stable rotaxane structure without any deterioration at high temperature.

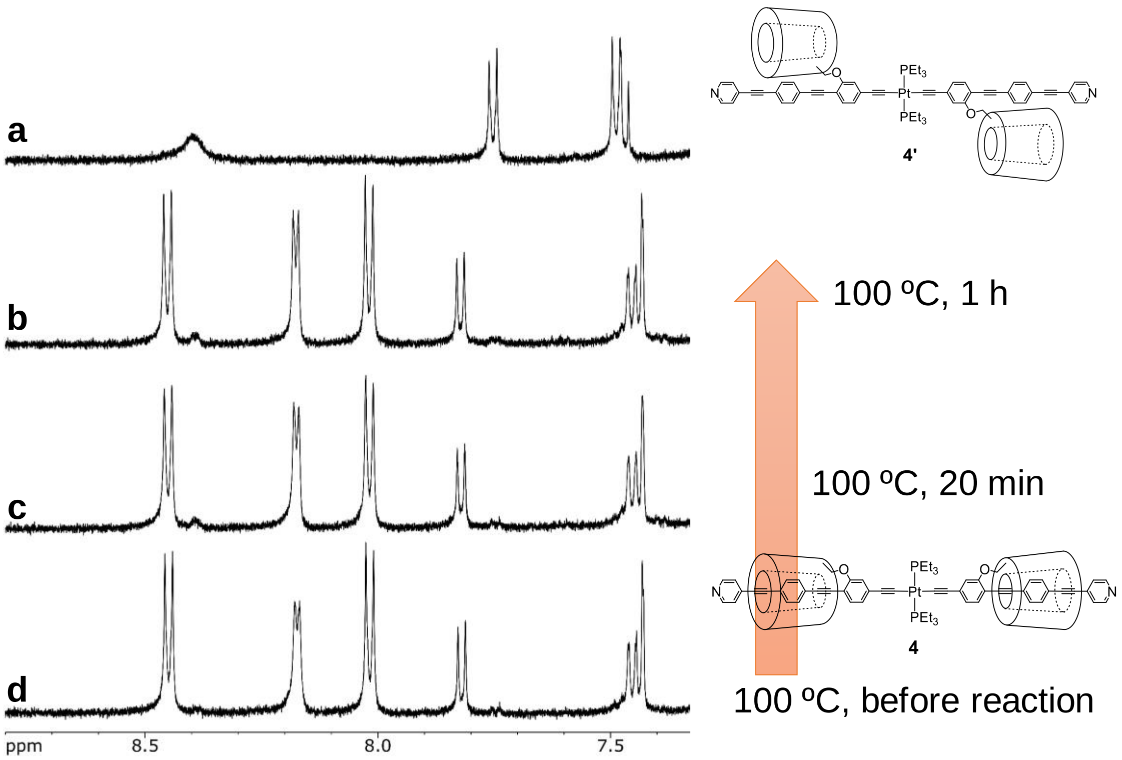

Supplementary Figure 3 Aromatic regions of the ^{1}H NMR spectra (500 MHz, toluene-d_8, r.t.); **a** uninsulated monomer **4′**, **b** after heating at 100 °C for 1 h, **c** after heating at 100 °C for 20 min, and **d** before heating of insulated monomer **4**.

Supplementary Note 4

Thermal analysis of bimetallopolymer

Thermogravimetry (TG) of bimetallopolymer **6** was conducted to examine its thermal stability. The bimetallopolymer in the solid state was stable below 300 °C under ambient air, while a drastic weight loss was observed above 300 °C (Supplementary Fig. 4).

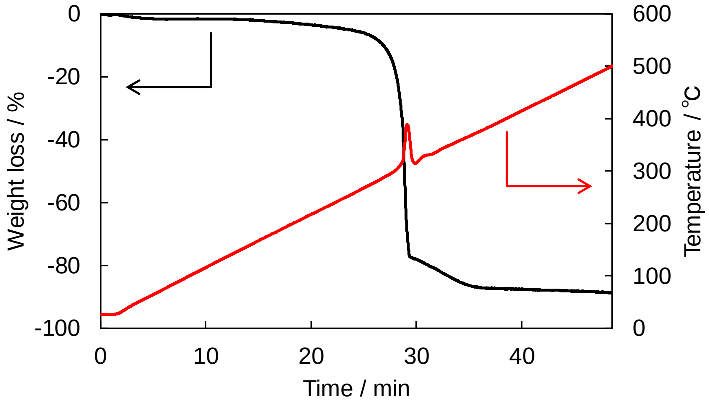

Supplementary Figure 4 Thermogravimetric analysis of bimetallopolymer **6** in the solid state.

Supplementary Note 5

Optical analyses

Optical analyses were conducted for the toluene solutions of monomers **4** and **5**, bimetallopolymer **6** and the depolymerizing mixtures (Supplementary Fig. 5–7). The depolymerizing mixture was commuted with the 1:1 molar mixture of **4** and **5**, which were the same components as the resultants after CO gas exposure to bimetallopolymer **6**. The absorption spectra after reaction with **6** and CO gas was corresponding with that of the mixture of **4** and **5**.

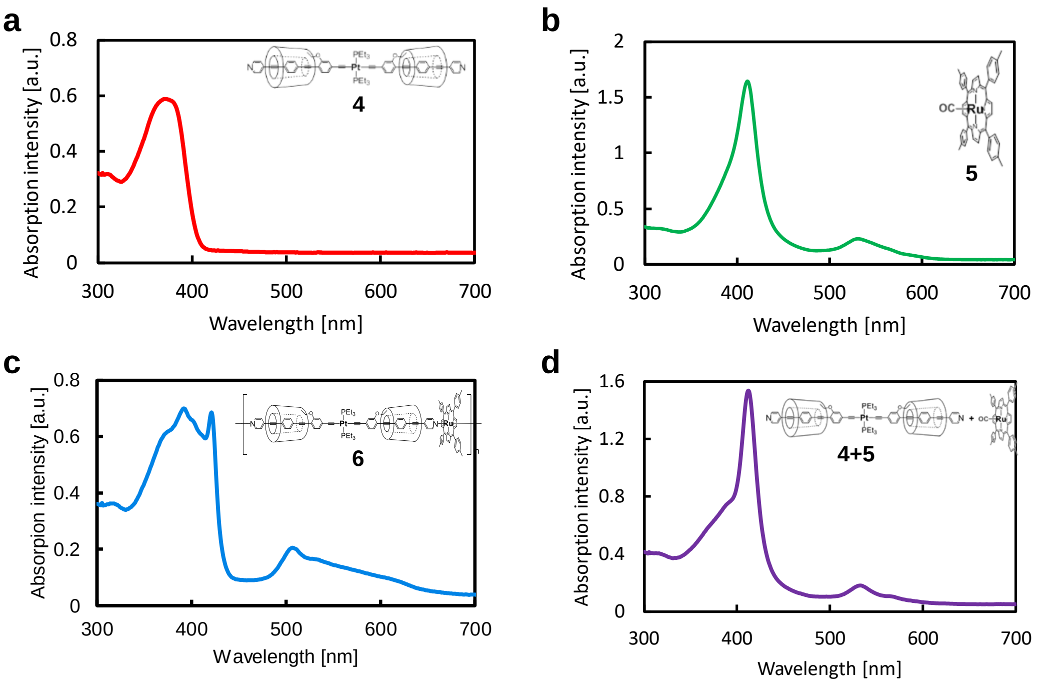

Supplementary Figure 5 Absorption spectra in dilute toluene solution; **a** **4**, **b** **5**, **c** **6**, and **d** depolymerizing mixture.

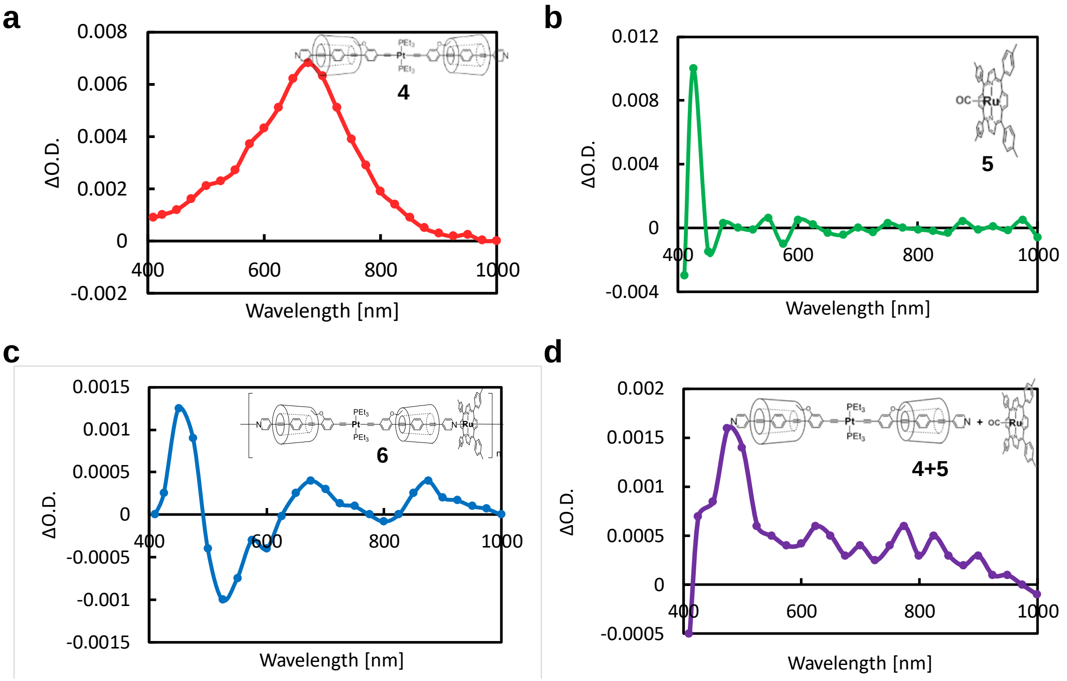

Supplementary Figure 6 Transient absorption spectra in a degassed atmosphere after the excitation at 337 nm; **a** **4** at 10 ns, **b** **5** at 1.5 ns, **c** **6** at 10 ns, and **d** depolymerizing mixture at 10 ns.

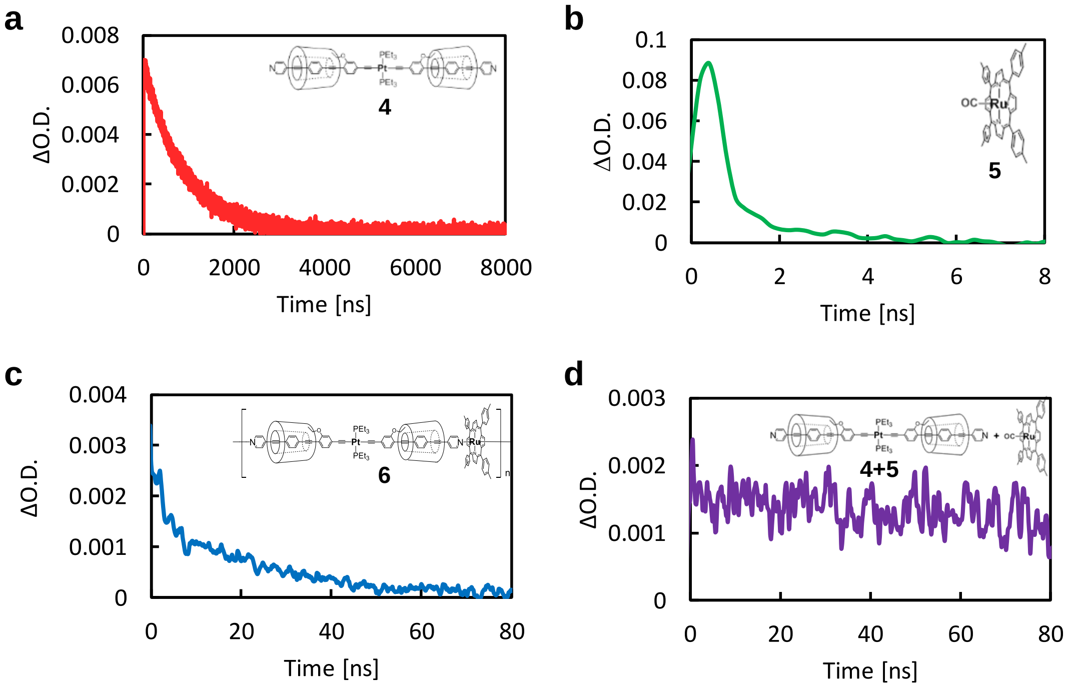

Supplementary Figure 7 Transient absorption decay in a degassed atmosphere after excitation at 337 nm; **a** **4** monitored at 675 nm, **b** **5** monitored at 425 nm, **c** **6** monitored at 450 nm, and **d** depolymerizing mixture monitored at 475 nm.

Supplementary Note 6

Concentration dependence of bimetallopolymer 6

Absorption spectra of bimetallopolymer 6 for various concentrations ranging from 3.3 × 10^{-3} mg/mL to 5.0 × 10^{-2} mg/mL and their maximum absorption wavelengths were independent of the diluted solution concentrations Supplementary Fig. 8). The results indicated that the bimetallopolymer did not form any assembled structures for diluted solution concentrations during our experiments.

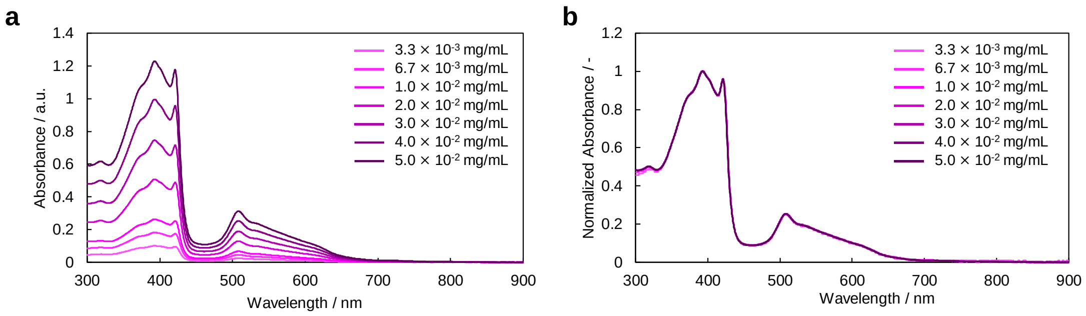

Supplementary Figure 8 Absorption spectra of bimetallopolymer **6** in toluene; **a** under various concentrations and **b** its normalized spectra.

Supplementary Note 7

Thermal effect of sensing reactions

The depolymerizing reaction was accelerated along with increasing the reaction temperature (Supplementary Fig. 9). Depolymerization was conducted under 1 atm CO gas according to the general procedure. SEC chromatogram of the resultant solution demonstrated that the complete depolymerizations needed 3 days, 4 hours, and 20 minutes under 60 °C, 80 °C, and 100 °C, respectively, while depolymerization incompletely proceeded after 10 hours under 60 °C. Stable bimetallopolymer was activated as a CO gas sensor by heating the solution.

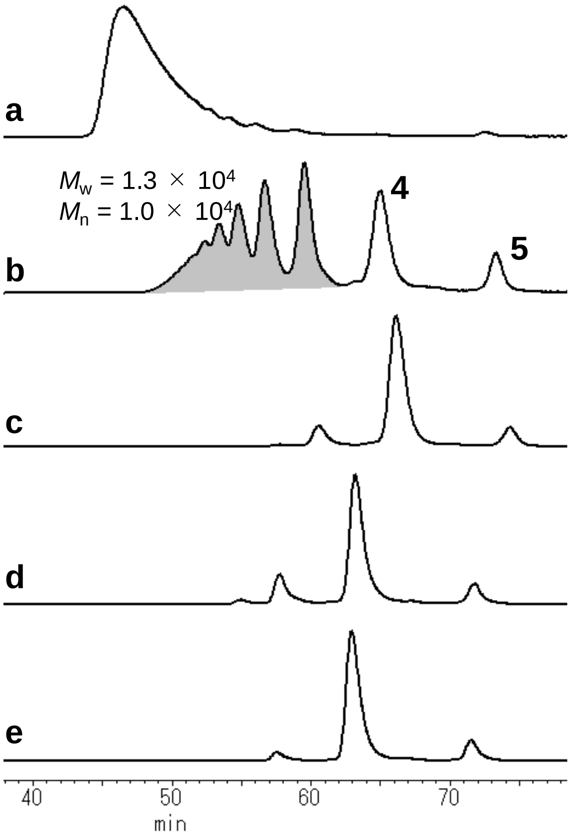

Supplementary Figure 9 SEC analyses (detected: UV 380 nm) of CO gas treatment under various temperature and reaction time; **a** before reaction, **b** 10 hours and **c** 3 days under 60 °C, **d** 4 hours under 80 °C, and **e** 20 min under 100 °C.

Supplementary Note 8

Recycling experiment

As shown in Supplementary Fig. 10b, bimetallopolymer successfully depolymerized to monomers **4** and **5** after the reaction with CO gas. Similarly, analysis with 254 nm detector indicated that Ru porphyrin **5** was successfully generated in the reaction mixture (Supplementary Fig. 10d). Subsequent UV irradiation provided repolymerizing bimetallopolymer **6** along with consuming the monomers as shown in Supplementary Fig. 10c. While the polymerization degree after repolymerization were slightly less than that before depolymerization, these results demonstrated that the reaction with CO gas for bimetallopolymer **6** afforded the monomers **4** and **5** without critical decompositions of each fragment.

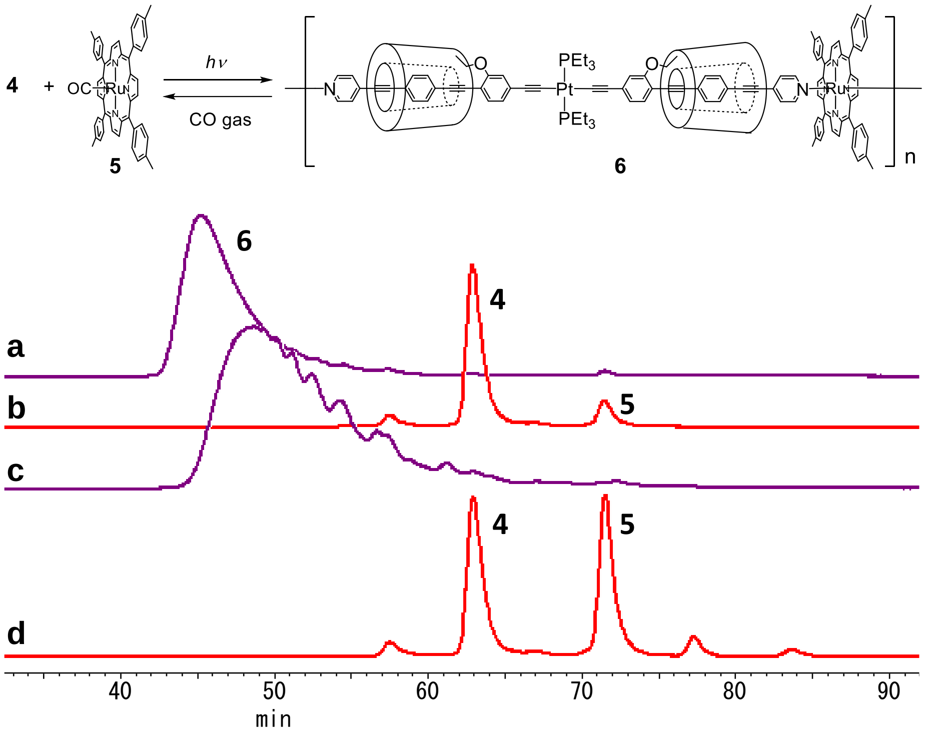

Supplementary Figure 10 SEC analyses of recycling experiment. Analyses of **a** bimetallopolymer **6** (before reaction), **b** after reaction with CO gas, and **c** after repolymerztion (detected: UV 380 nm). **d** Analysis (detected: UV 254 nm) in **b**: after depolymerization.

Supplementary Note 9

Gas selectivity

Supplementary Fig. 11 displayed the emission spectra of each resultant mixture with polymer and monomers. In Fig. 3f, the emission intensities at 557 nm was indicated against each target gas.

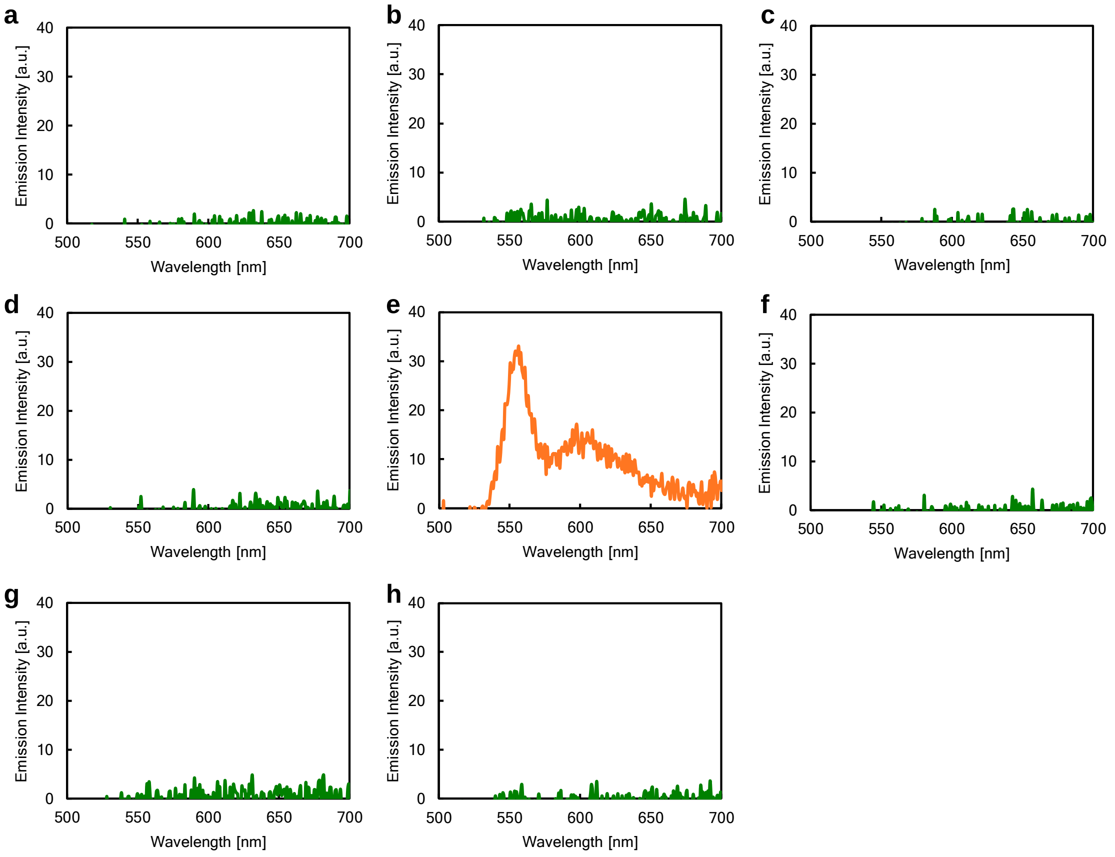

Supplementary Figure 11 Emission spectra of the dilute and deoxygenated toluene solutions after reaction with various gases; **a** N_2, **b** O_2, **c** H_2, **d** CO_2, **e** CO, **f** CH_4, **g** C_2H_4, and **h** 1%v/v H_2S in N_2 (concentration: 10^{-2} mg/mL, excitation at 365 nm).

A mixed gas experiment was conducted by using ambient air (a mixture of N_2, O_2, and CO_2) as one of the mixed gases. The bimetallopolymer **6** responded to 1% CO gas under mixed gas with ambient air to display phosphorescence (Supplementary Fig. 12 and 13). The results indicated that the responsiveness of the bimetallopolymer to CO gas was not affected even under the mixed gas condition.

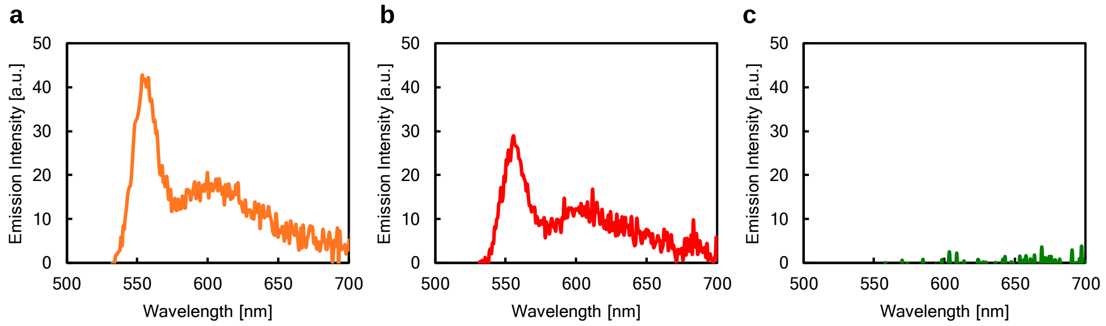

Supplementary Figure 12 Emission spectra of the dilute and deoxygenated toluene solutions after reaction with various gases; **a** 1% v/v CO in N_2, **b** 1% v/v CO in air, and **c** air (concentration: 10^{-2} mg/mL, excitation at 365 nm).

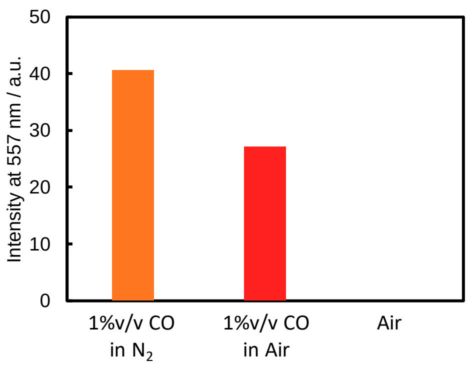

Supplementary Figure 13 Emission intensities after depolymerization of the bimetallopolymer **6** with various gases under UV irradiation.

Supplementary Note 10

Concentration dependence and tunable thresholds for dual self-controlling system

Supplementary Fig. 14 displayed the emission spectra of each resultant mixture. In Fig. 4b, the emission intensities at 557 nm were plotted against CO gas concentrations. Supplementary Fig. 15 demonstrated the two thresholds with the linear concentration scales.

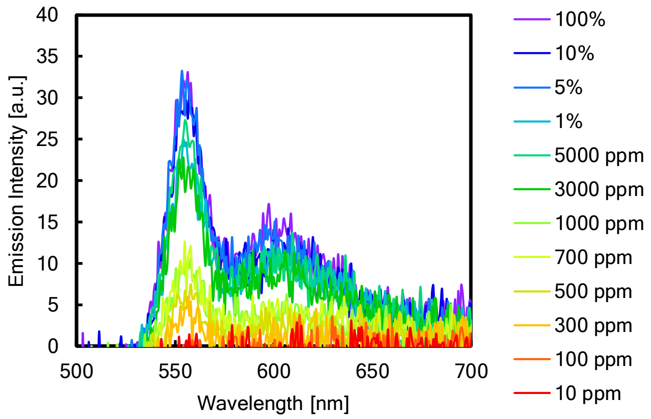

Supplementary Figure 14 Emission spectra of the dilute and deoxygenated toluene solutions after reaction at 100 °C for 10 min with various concentrations of CO gas (concentration: 10^{-2} mg/mL, excitation at 365 nm).

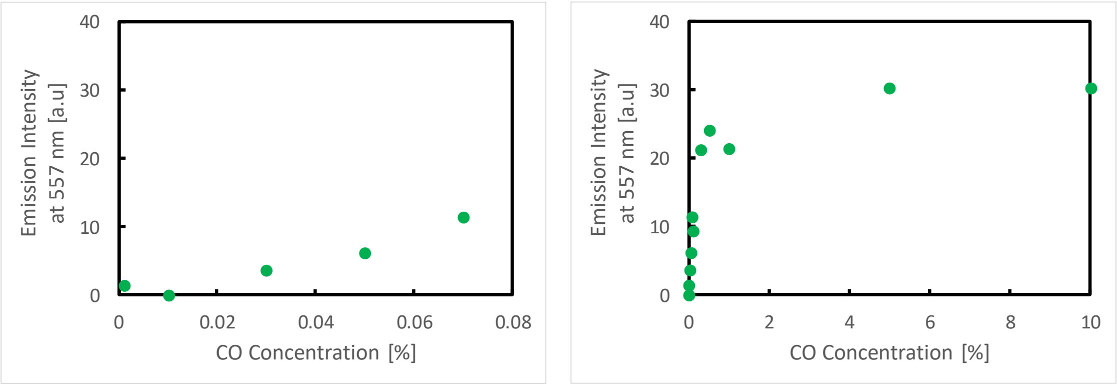

Supplementary Figure 15 Concentration-dependences of emission intensities at 577 nm in Supplementary Fig. 14 with linear concentration scales.

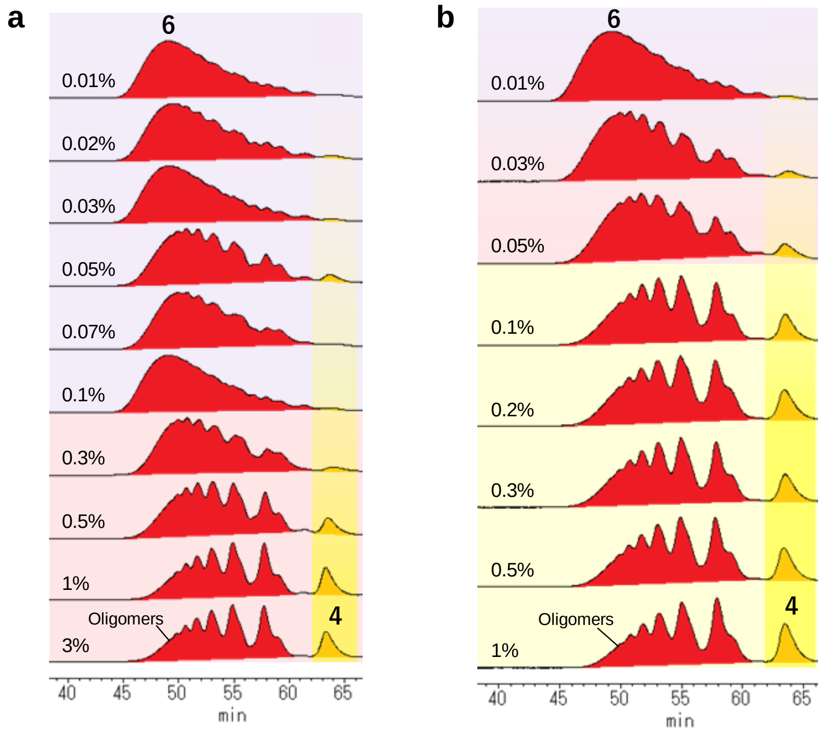

Supplementary Figure 16 SEC profiles (UV detector, 380 nm) after reaction with various concentrations of CO gas; **a** at 100 °C for 3 min and **b** at 90 °C for 10 min.

Supplementary Fig. 17 displayed the emission spectra of each resultant mixture. In Fig. 6, the emission intensities at 557 nm, which was determined with the average value of 5 adjacent points between 556 nm to 559 nm, were plotted against CO gas concentrations.

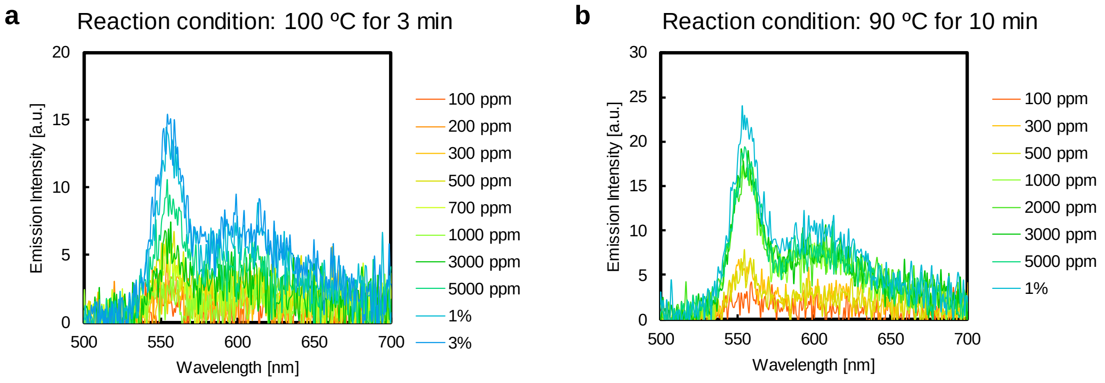

Supplementary Figure 17 Emission spectra of the dilute and deoxygenated toluene solutions after reaction with various concentrations of CO gas; **a** at 100 °C for 3 min and **b** at 90 °C for 10 min (concentration: 10^{-2} mg/mL, excitation at 365 nm).

Supplementary Note 11

ONIOM calculation of optimized 4 structure

The calculated structure of **4** shown in Supplementary Fig. 2 was determined by ONIOM[4-6] calculations. In the calculation, the molecular system was divided into two layers above C (sp^2)—O bonds. The high layers were assigned to the conjugated backbones, involving the phenylene ethynylene moieties and the Pt complex, for B3LYP/Lanl2DZ calculation. The low layers containing PM α-CD parts were calculated on semi-empirical molecular orbital calculation using PM6 method. All calculations were performed with the Gaussian 09.[7] Details of the optimized geometries of **4** is presented in Supplementary Table 1.

Supplementary Table 1. Optimized structural coordinates of **4**.

Center	Atomic	Coordinations (Angstroms)		
Number	Symbol	X	Y	Z
1	O	-7.3677	2.5986	-0.0744
2	O	-9.2366	4.6245	0.4046
3	O	-12.349	6.5871	0.3508
4	O	-11.4932	6.1116	-2.4706
5	O	-10.9874	3.0542	-2.3105
6	O	-10.6062	2.339	-4.4503
7	O	-13.556	3.4604	-2.6897
8	O	-14.3993	0.999	-4.2036
9	O	-8.3538	0.0421	-4.348
10	O	-12.184	-0.9354	-3.7792
11	O	-10.5917	-2.4117	-4.5645
12	O	-14.1698	-2.7775	-4.2269
13	O	-12.9952	-5.1063	-2.8437
14	O	-8.2581	-4.2129	-3.6422
15	O	-10.7027	-3.9429	-1.2123
16	O	-8.7962	-3.5377	-0.0008
17	O	-10.5328	-6.7457	-0.4707
18	O	-10.7259	-6.1512	2.4434
19	O	-7.8624	-2.8251	2.8489
20	O	-11.4986	-3.1577	2.3094
21	O	-11.2651	-2.7949	4.585
22	O	-14.072	-3.3883	2.3833
23	O	-14.8242	-1.0551	4.1413
24	O	-10.7526	-0.6449	6.7329
25	O	-12.3064	0.6765	3.904
26	O	-10.4147	1.9228	4.2905
27	O	-13.8798	2.5134	5.1628
28	O	-13.2769	4.798	3.4519
29	O	-8.5342	4.084	3.455
30	O	-11.2619	4.1249	1.4298
31	C	-14.478	0.1068	0.0691
32	C	-13.7858	1.3473	0.0331
33	C	-12.395	1.3875	-0.1096
34	C	-11.6437	0.1868	-0.2316
35	C	-12.3351	-1.0539	-0.1729
36	C	-13.7242	-1.0946	-0.0218
37	C	-10.2246	0.2185	-0.4093
38	C	-9.0042	0.2374	-0.5614
39	C	-7.585	0.2341	-0.69
40	C	-6.7893	1.4021	-0.4621
41	C	-5.3859	1.3765	-0.5247
42	C	-4.697	0.1717	-0.828
43	C	-5.4832	-0.9908	-1.0805
44	C	-6.8783	-0.9592	-1.0094
45	C	-10.4092	5.1834	1.0072
46	C	-11.0962	6.0337	-0.0893
47	C	-11.4496	5.2076	-1.3387
48	C	-10.3334	4.2307	-1.7699
49	C	-9.4747	3.6435	-0.6263
50	C	-8.0664	3.3136	-1.1321
51	C	-12.2152	7.6525	1.3168
52	C	-12.7782	6.7402	-2.6628
53	C	-11.4345	3.1836	-3.6663
54	C	-12.9124	2.7393	-3.7585
55	C	-13.1158	1.2201	-3.5808
56	C	-12.0627	0.3931	-4.3602
57	C	-10.6297	0.9199	-4.1126
58	C	-9.5654	0.3466	-5.0574
59	C	-14.9829	3.6114	-2.8626
60	C	-15.1645	-0.0715	-3.6064
61	C	-7.5326	1.1802	-4.0299
62	C	-11.9662	-2.0322	-4.661
63	C	-12.8135	-3.2467	-4.2143
64	C	-12.4249	-3.7786	-2.8167
65	C	-10.8895	-3.9003	-2.6512
66	C	-10.1383	-2.6691	-3.2013
67	C	-8.6248	-2.8541	-3.3456
68	C	-15.1553	-3.8247	-4.381
69	C	-13.2228	-5.6853	-1.5418
70	C	-8.4353	-4.5765	-5.0284
71	C	-9.4853	-4.5405	-0.7429
72	C	-9.8195	-5.7039	0.2228
73	C	-10.7766	-5.2294	1.332
74	C	-10.3009	-3.9143	1.9804

75	C	-9.5713	-2.9154	1.06	129	H	-9.1252	-1.3315	2.4863
76	C	-8.569	-2.0654	1.8569	130	H	-9.291	-6.9755	-2.1429
77	C	-9.7118	-7.5676	-1.3248	131	H	-10.4341	-8.2977	-1.7114
78	C	-11.5438	-7.3274	2.2746	132	H	-8.928	-8.063	-0.745
79	C	-6.797	-3.6398	2.3155	133	H	-12.6015	-7.0515	2.3081
80	C	-12.054	-3.44	3.592	134	H	-11.266	-7.9396	3.1393
81	C	-13.4838	-2.8577	3.5873	135	H	-11.2968	-7.8333	1.3325
82	C	-13.5226	-1.3133	3.5646	136	H	-7.1649	-4.2689	1.497
83	C	-12.45	-0.6592	4.4657	137	H	-6.4987	-4.2427	3.1797
84	C	-11.0812	-1.3654	4.3899	138	H	-5.9776	-2.9994	1.9766
85	C	-10.0933	-0.9755	5.4995	139	H	-9.3511	-1.7799	5.6549
86	C	-15.5167	-3.4102	2.4115	140	H	-9.5783	-0.0212	5.2508
87	C	-15.3685	0.2443	3.83	141	H	-15.8622	-4.1744	3.1128
88	C	-11.1938	-1.7922	7.4907	142	H	-15.7732	-3.6717	1.3806
89	C	-11.7212	1.6489	4.7765	143	H	-15.9125	-2.4221	2.6847
90	C	-12.5761	2.9386	4.7378	144	H	-16.2255	0.3166	4.5057
91	C	-12.6126	3.5252	3.3096	145	H	-15.6769	0.2732	2.7824
92	C	-11.1615	3.7853	2.8451	146	H	-14.6305	1.035	4.049
93	C	-10.2928	2.513	2.965	147	H	-11.8389	-1.348	8.2553
94	C	-8.7866	2.7842	2.8607	148	H	-10.3272	-2.2843	7.9412
95	C	-14.6804	3.5651	5.7474	149	H	-11.7488	-2.4909	6.8529
96	C	-14.1127	5.1369	2.3238	150	H	-8.4556	2.7736	1.7994
97	C	-7.3836	4.7395	2.8854	151	H	-15.648	3.0724	5.8897
98	H	-8.1117	2.7133	-2.0689	152	H	-14.7582	4.4136	5.0555
99	H	-7.4596	4.2286	-1.2729	153	H	-14.2501	3.8718	6.7044
100	H	-13.2261	8.0765	1.3464	154	H	-14.4877	6.1298	2.5883
101	H	-11.4927	8.402	0.9847	155	H	-14.9299	4.4182	2.2303
102	H	-11.9469	7.2322	2.2928	156	H	-13.5006	5.1782	1.4067
103	H	-12.5876	7.4395	-3.4828	157	H	-7.3924	5.7175	3.3758
104	H	-13.0798	7.2674	-1.749	158	H	-7.4959	4.8327	1.7955
105	H	-13.5164	5.9806	-2.9433	159	H	-6.4749	4.1857	3.1371
106	H	-9.8421	-0.6601	-5.4403	160	H	-14.3475	2.2745	0.1042
107	H	-9.3653	1.0383	-5.8956	161	H	-11.8792	2.3498	-0.151
108	H	-15.2964	4.0246	-1.8984	162	H	-11.762	-1.9858	-0.2257
109	H	-15.4552	2.6383	-3.0511	163	H	-14.2331	-2.054	0.0476
110	H	-15.1881	4.3046	-3.6826	164	H	-4.8242	2.2845	-0.3202
111	H	-15.5451	0.2453	-2.6318	165	H	-7.455	-1.8745	-1.1911
112	H	-14.5444	-0.9788	-3.5111	166	H	-9.9974	5.7646	1.8601
113	H	-15.9714	-0.2268	-4.328	167	H	-10.4071	6.8666	-0.3735
114	H	-7.1996	1.6793	-4.9431	168	H	-12.4248	4.6719	-1.2197
115	H	-6.6912	0.7232	-3.4921	169	H	-9.7099	4.7158	-2.5583
116	H	-8.0797	1.8799	-3.3766	170	H	-9.987	2.765	-0.1603
117	H	-8.2155	-2.154	-4.1032	171	H	-11.2578	4.2022	-4.0774
118	H	-8.1143	-2.6815	-2.3669	172	H	-13.3386	3.0565	-4.7407
119	H	-14.9911	-4.6193	-3.6404	173	H	-13.1326	0.9305	-2.5055
120	H	-15.1166	-4.2205	-5.3991	174	H	-12.3206	0.3624	-5.4397
121	H	-16.095	-3.2939	-4.1961	175	H	-10.3387	0.8005	-3.0421
122	H	-13.6288	-6.6718	-1.7848	176	H	-12.1073	-1.7659	-5.7271
123	H	-13.9376	-5.0838	-0.9757	177	H	-12.6879	-4.0671	-4.9637
124	H	-12.2646	-5.777	-1.0041	178	H	-12.874	-3.1707	-2.0014
125	H	-7.6726	-4.0789	-5.6348	179	H	-10.5218	-4.8489	-3.1018
126	H	-9.441	-4.307	-5.3732	180	H	-10.3659	-1.762	-2.5867
127	H	-8.2891	-5.6607	-5.0221	181	H	-8.7857	-4.8074	-1.5639
128	H	-7.8584	-1.5429	1.1955	182	H	-8.8828	-6.1149	0.6622

183	H	-11.819	-5.1327	0.9468	237	C	11.3998	-5.2015	-1.4234
184	H	-9.7095	-4.127	2.9097	238	H	9.6615	-4.6823	-2.6334
185	H	-10.3348	-2.2694	0.5431	239	C	12.3601	-1.4063	-0.1308
186	H	-11.9879	-4.518	3.8531	240	C	12.3085	1.0361	-0.1532
187	H	-14.0401	-3.2269	4.4827	241	O	11.2156	-4.1688	1.3645
188	H	-13.4639	-0.9167	2.5258	242	C	11.043	-6.049	-0.1893
189	H	-12.8172	-0.5768	5.5151	243	H	9.9451	-5.8115	1.7646
190	H	-10.6146	-1.212	3.3827	244	C	11.3918	-3.1375	-3.715
191	H	-11.5472	1.2603	5.8074	245	O	11.4401	-6.0849	-2.5715
192	H	-12.1636	3.6876	5.4528	246	H	12.3769	-4.6716	-1.2946
193	H	-13.1584	2.858	2.6086	247	C	13.751	-1.3732	0.0125
194	H	-10.7093	4.6357	3.4081	248	H	11.841	-2.366	-0.1885
195	H	-10.6209	1.7423	2.2284	249	C	13.6978	1.0695	-0.0015
196	H	-8.2153	2.0455	3.4535	250	H	11.7387	1.9707	-0.1903
197	H	-4.9747	-1.921	-1.3214	251	C	11.1148	-3.8535	2.7856
198	C	-15.9027	0.0674	0.1921	252	O	12.2934	-6.6148	0.2412
199	C	-17.1267	0.0317	0.2915	253	H	10.3504	-6.8736	-0.4889
200	C	-18.5505	-0.0095	0.4091	254	O	10.5666	-2.2769	-4.4846
201	C	-19.2545	-1.2417	0.4321	255	C	12.8714	-2.6974	-3.7995
202	C	-19.3184	1.18	0.5076	256	H	11.2114	-4.1485	-4.1433
203	C	-20.6535	-1.2304	0.5492	257	C	12.7226	-6.7153	-2.7749
204	H	-18.7167	-2.1815	0.3609	258	C	14.4473	-0.1358	0.0693
205	C	-20.7146	1.0878	0.6212	259	H	14.3095	-2.3033	0.0681
206	H	-18.831	2.1495	0.4968	260	H	14.2099	2.0258	0.0844
207	N	-21.3902	-0.0917	0.6429	261	C	12.5664	-3.6078	3.2561
208	H	-21.2153	-2.1606	0.57	262	C	10.2507	-2.5803	2.9262
209	H	-21.3246	1.9842	0.6985	263	H	10.6587	-4.712	3.3332
210	C	-3.2682	0.1286	-0.8771	264	C	12.1548	-7.7036	1.1802
211	C	-2.0251	0.0953	-0.8926	265	C	10.5959	-0.8636	-4.1238
212	Pt	-0.022	0.0307	-0.895	266	O	13.5119	-3.4375	-2.7419
213	C	1.9933	-0.0451	-0.8871	267	C	13.081	-1.1821	-3.5981
214	C	3.2364	-0.0973	-0.869	268	H	13.2968	-3.001	-4.7864
215	C	4.6667	-0.1493	-0.8227	269	H	12.5291	-7.3989	-3.6073
216	C	5.3505	-1.3614	-0.54	270	H	13.022	-7.26	-1.8707
217	C	5.4567	1.0135	-1.0583	271	H	13.464	-5.9538	-3.0417
218	C	6.7542	-1.3943	-0.4803	272	C	15.8721	-0.1032	0.1933
219	H	4.7865	-2.2711	-0.3493	273	O	13.2237	-4.8861	3.3802
220	C	6.8521	0.9751	-0.9899	274	C	12.5312	-3.0431	4.6931
221	H	4.952	1.9495	-1.2838	275	H	13.1168	-2.9329	2.566
222	O	7.3273	-2.5993	-0.1133	276	O	10.3745	-2.0112	4.2606
223	C	7.5541	-0.2258	-0.6903	277	C	8.7434	-2.8447	2.818
224	H	7.4322	1.8909	-1.1578	278	H	10.5813	-1.7993	2.2018
225	C	8.0239	-3.2989	-1.1829	279	H	13.1658	-8.1277	1.2051
226	C	8.9735	-0.2367	-0.5628	280	H	11.4347	-8.4451	0.8254
227	C	9.4306	-3.6434	-0.6827	281	H	11.8804	-7.3078	2.1646
228	H	8.0718	-2.683	-2.1093	282	C	12.0308	-0.3385	-4.3635
229	H	7.4133	-4.209	-1.3392	283	C	9.5335	-0.2707	-5.0585
230	C	10.1937	-0.2249	-0.4107	284	H	10.3059	-0.7607	-3.0514
231	O	9.1887	-4.6418	0.3303	285	C	14.9381	-3.5921	-2.9172
232	C	10.287	-4.2132	-1.8369	286	O	14.3649	-0.9564	-4.2184
233	H	9.9463	-2.7754	-0.201	287	H	13.0998	-0.9095	-2.5184
234	C	11.6131	-0.2011	-0.2327	288	C	17.0962	-0.0731	0.2937
235	C	10.3592	-5.2163	0.9226	289	C	14.0586	-5.2133	2.248
236	O	10.9447	-3.0296	-2.3573	290	O	13.8365	-2.6303	5.1254

291	C	11.6819	-1.7505	4.7514	345	H	21.192	2.1015	0.6028
292	H	12.1149	-3.8013	5.3961	346	H	21.2872	-2.045	0.6773
293	O	8.4868	-4.153	3.3915	347	O	14.7977	0.9485	4.1595
294	H	8.4123	-2.8157	1.7572	348	C	13.466	2.7663	3.6355
295	H	8.175	-2.1138	3.423	349	H	13.4367	0.8431	2.5422
296	O	12.1574	0.9799	-3.7608	350	O	11.2469	2.6979	4.6321
297	H	12.2884	-0.2911	-5.4425	351	C	10.0662	0.8693	5.5162
298	O	8.3218	0.023	-4.3444	352	H	10.5893	1.1383	3.4037
299	H	9.8124	0.7424	-5.4225	353	C	10.8751	3.9304	-2.583
300	H	9.3325	-0.9462	-5.9096	354	C	8.6061	2.9036	-3.2928
301	H	15.2497	-4.022	-1.9597	355	H	10.344	1.7932	-2.5538
302	H	15.4147	-2.6182	-3.0902	356	C	15.1396	3.8672	-4.3176
303	H	15.1402	-4.2731	-3.7481	357	O	12.9848	5.1316	-2.757
304	C	15.1352	0.101	-3.6047	358	H	12.8572	3.1827	-1.9468
305	C	18.5201	-0.0383	0.4125	359	C	15.3357	-0.3483	3.8272
306	H	14.4261	-6.2128	2.4975	360	O	14.0566	3.3138	2.4402
307	H	14.8812	-4.4991	2.167	361	C	12.0393	3.3558	3.6505
308	H	13.4478	-5.2357	1.3294	362	H	14.0245	3.1179	4.5367
309	C	14.6316	-3.6943	5.6952	363	O	10.7236	0.5148	6.7438
310	O	12.2714	-0.7669	3.8944	364	H	9.328	1.6749	5.685
311	H	11.509	-1.3774	5.7882	365	H	9.5463	-0.078	5.2514
312	C	7.3358	-4.7972	2.8099	366	O	10.6891	3.9493	-1.1435
313	C	11.9436	2.0919	-4.6243	367	H	10.5107	4.8877	-3.0174
314	C	7.4991	-1.1198	-4.0473	368	O	8.243	4.2682	-3.5668
315	H	15.5148	-0.2328	-2.6354	369	H	8.1938	2.2173	-4.0614
316	H	14.5195	1.0096	-3.4949	370	H	8.096	2.7168	-2.3164
317	H	15.9426	0.2638	-4.3242	371	H	14.979	4.6508	-3.5646
318	C	19.2281	1.1911	0.452	372	H	15.1016	4.2788	-5.3294
319	C	19.2837	-1.2316	0.4958	373	H	16.0774	3.33	-4.1416
320	H	15.6014	-3.2081	5.8456	374	C	13.2177	5.6871	-1.4457
321	H	14.7061	-4.5328	4.9909	375	H	16.1927	-0.4353	4.5013
322	H	14.199	-4.0129	6.6472	376	H	15.6436	-0.3618	2.7792
323	C	12.4215	0.5588	4.4777	377	H	14.5942	-1.1389	4.034
324	H	7.3418	-5.7826	3.2856	378	C	15.5015	3.3288	2.469
325	H	7.4498	-4.8743	1.719	379	O	11.4821	3.0987	2.3632
326	H	6.4278	-4.2455	3.0684	380	H	11.9789	4.4294	3.9301
327	O	10.5704	2.475	-4.5206	381	C	11.1701	1.6468	7.5212
328	C	12.7957	3.2956	-4.158	382	C	9.475	4.545	-0.6635
329	H	12.083	1.8427	-5.6947	383	C	8.4227	4.6547	-4.9465
330	H	7.1671	-1.6026	-4.9695	384	H	13.6257	6.6766	-1.6727
331	H	6.6575	-0.6717	-3.5026	385	H	13.9326	5.0739	-0.8924
332	H	8.045	-1.8313	-3.4059	386	H	12.2613	5.772	-0.9037
333	C	20.627	1.1735	0.5696	387	H	15.8503	4.0795	3.183
334	H	18.6935	2.1336	0.3928	388	H	15.7593	3.6065	1.4426
335	C	20.6802	-1.1457	0.6113	389	H	15.8928	2.3344	2.7255
336	H	18.7931	-2.1992	0.4721	390	C	10.2882	3.8671	2.0483
337	C	13.4973	1.2223	3.5873	391	H	11.8128	1.1864	8.2783
338	C	11.0562	1.2727	4.4133	392	H	10.3058	2.1353	7.9798
339	H	12.7883	0.4577	5.5256	393	H	11.7288	2.3535	6.8955
340	C	10.1191	2.7112	-3.153	394	O	8.7808	3.5329	0.0613
341	O	14.1502	2.8215	-4.1789	395	C	9.8153	5.6896	0.3223
342	C	12.4099	3.8057	-2.7515	396	H	8.7767	4.8296	-1.4796
343	H	12.6729	4.1288	-4.8936	397	H	7.6592	4.1699	-5.562
344	N	21.3597	0.0312	0.6488	398	H	9.4279	4.388	-5.295

399	H	8.28	5.7391	-4.9218
400	C	10.7701	5.1909	1.4229
401	C	9.5528	2.8882	1.1109
402	H	9.6985	4.0664	2.9816
403	O	10.5342	6.7393	-0.3533
404	H	8.8809	6.0979	0.7689
405	O	10.7235	6.0934	2.5502
406	H	11.812	5.0961	1.0359
407	C	8.546	2.0301	1.8932
408	H	10.3126	2.2472	0.5826
409	C	9.7172	7.5823	-1.1906
410	C	11.548	7.268	2.4025
411	O	7.8443	2.7758	2.8993
412	H	7.8319	1.5238	1.2231
413	H	9.0981	1.2819	2.5092
414	H	9.2909	7.0082	-2.0186
415	H	10.4438	8.3144	-1.5652
416	H	8.938	8.0724	-0.6003
417	H	12.6042	6.9858	2.432
418	H	11.2728	7.8663	3.2778
419	H	11.3044	7.7917	1.4694
420	C	6.7865	3.61	2.3814
421	H	7.1602	4.2513	1.575
422	H	6.4936	4.1992	3.2569
423	H	5.9616	2.9835	2.0303
424	P	0.0738	0.0855	-3.3082
425	P	0.0655	0.0483	1.522
426	C	-1.6118	0.1304	-4.1832
427	C	-1.6696	-0.3339	-5.6525
428	H	-1.9754	1.1609	-4.0815
429	H	-2.2715	-0.4829	-3.5611
430	H	-2.7066	-0.2749	-6.009
431	H	-1.0543	0.2838	-6.315
432	H	-1.3488	-1.3771	-5.7607
433	C	1.0196	-1.4324	-3.9571
434	C	1.7576	-1.3048	-5.3064
435	H	0.289	-2.252	-3.9878
436	H	1.7341	-1.6686	-3.1623
437	H	2.2742	-2.2484	-5.5272
438	H	1.079	-1.0946	-6.1399
439	H	2.5193	-0.5169	-5.2736
440	C	1.0284	1.6467	-3.8289
441	C	0.8617	2.1533	-5.2761
442	H	2.0796	1.4403	-3.5947
443	H	0.7024	2.4193	-3.1222
444	H	1.4434	3.0759	-5.406
445	H	1.2182	1.4303	-6.0172
446	H	-0.1839	2.392	-5.5039
447	C	1.0048	-1.4811	2.1515
448	C	1.7457	-1.3702	3.501
449	H	1.7179	-1.7123	1.354
450	H	0.2708	-2.2976	2.1762
451	H	2.2572	-2.3189	3.7129
452	H	2.5134	-0.5877	3.4747
453	H	1.0701	-1.1633	4.3377
454	C	1.0234	1.6005	2.063
455	C	0.856	2.0867	3.5172
456	H	0.6995	2.3832	1.3669
457	H	2.0743	1.3943	1.8282
458	H	1.44	3.0054	3.6624
459	H	-0.1895	2.3247	3.7478
460	H	1.2093	1.3517	4.2483
461	C	-1.6215	0.0846	2.3935
462	C	-1.6812	-0.3981	3.857
463	H	-2.283	-0.5188	1.7643
464	H	-1.9836	1.1173	2.307
465	H	-2.7188	-0.3406	4.2132
466	H	-1.363	-1.443	3.9538
467	H	-1.0661	0.2107	4.5282

Supplementary Figures

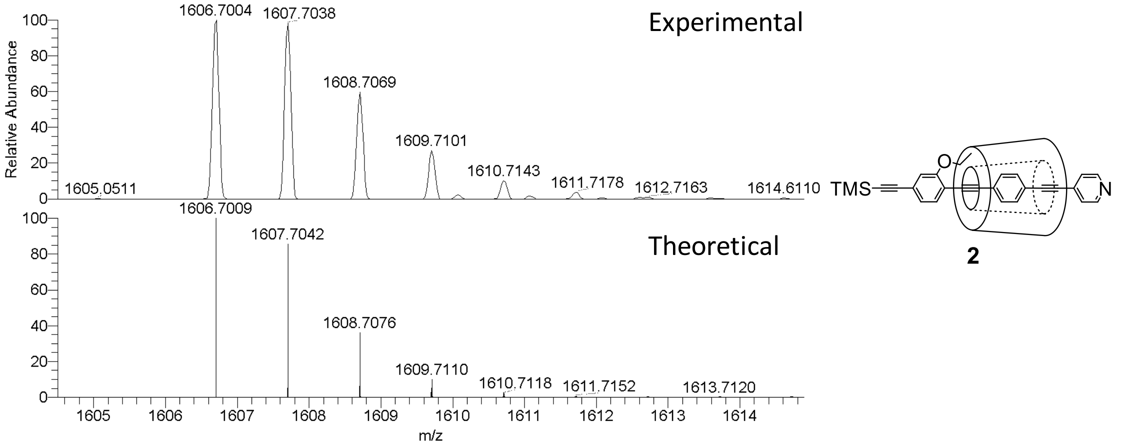

Supplementary Figure 18 MALDI TOF-MS spectrum of **2**

^{1}H NMR spectrum of **2**

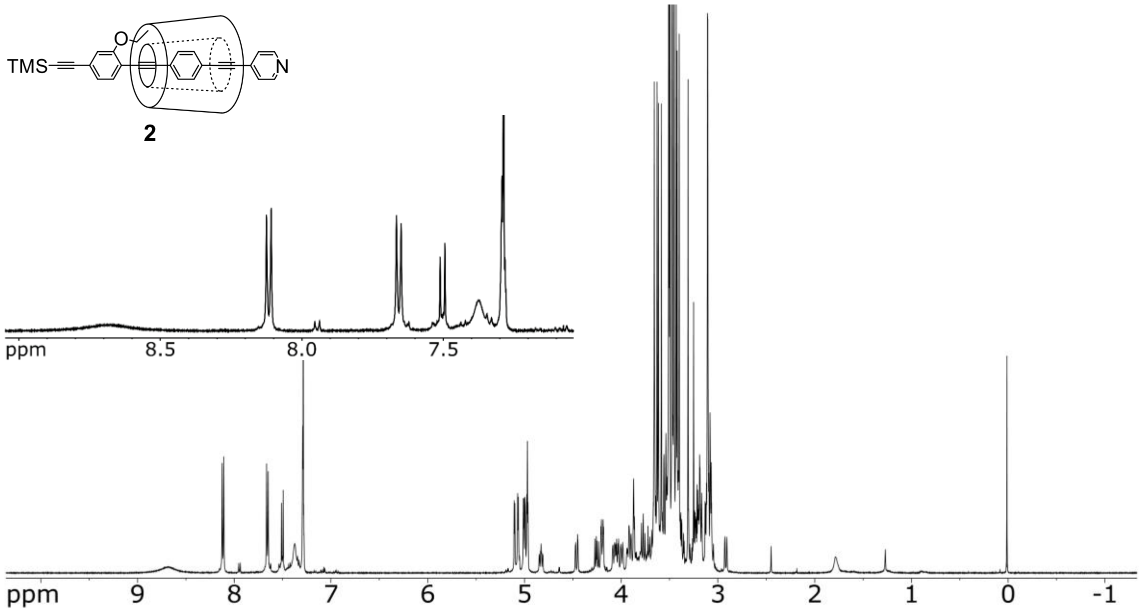

Supplementary Figure 19 ^{1}H NMR spectrum (500 MHz, $CDCl_3$) of **2**

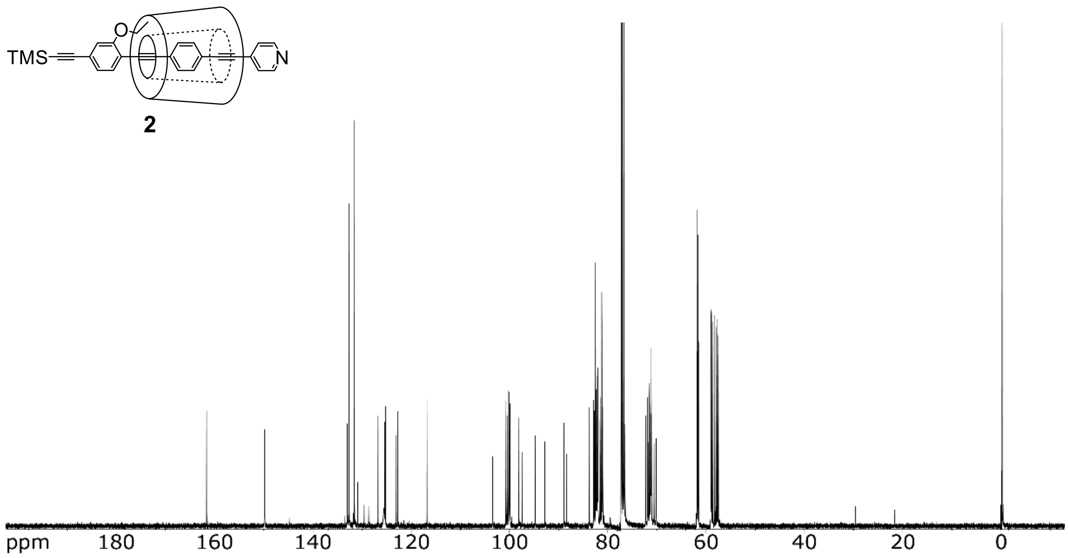

Supplementary Figure 20 ^{13}C NMR spectrum (126 MHz, $CDCl_3$) of **2**

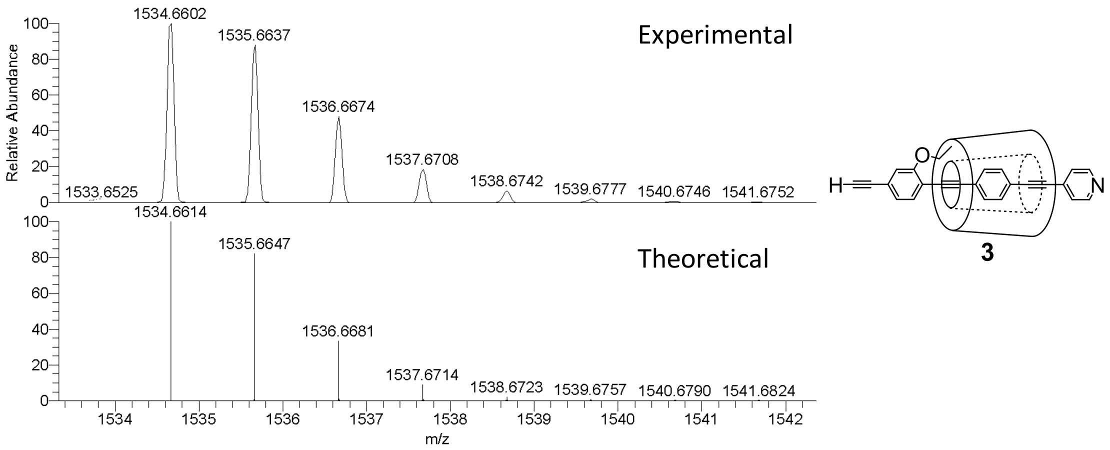

Supplementary Figure 21 MALDI TOF-MS spectrum of **3**

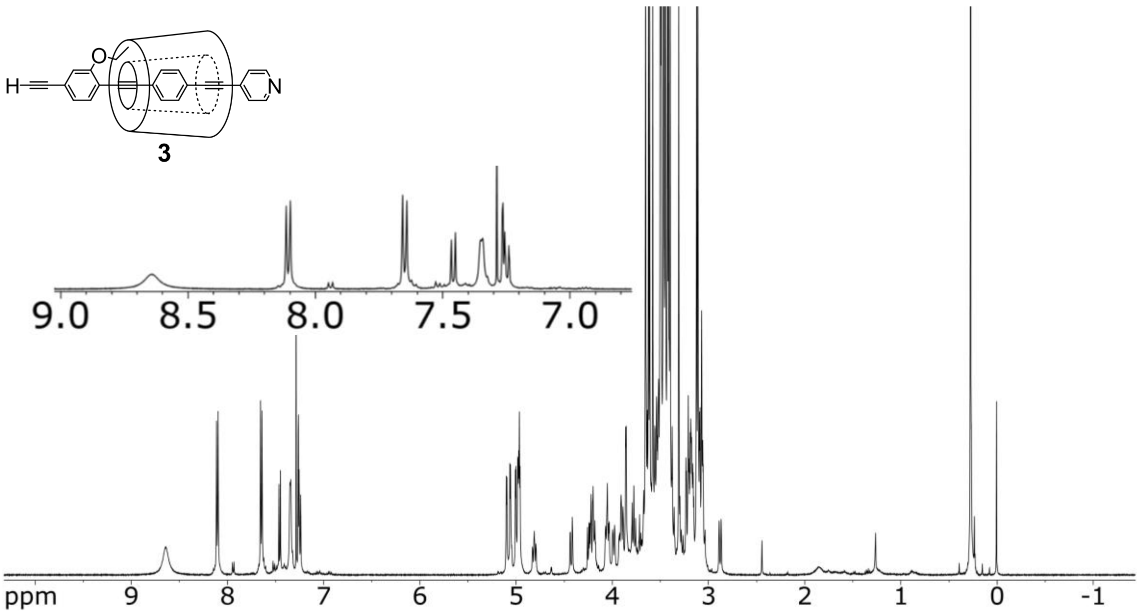

Supplementary Figure 22 ^{1}H NMR spectrum (500 MHz, $CDCl_3$) of **3**

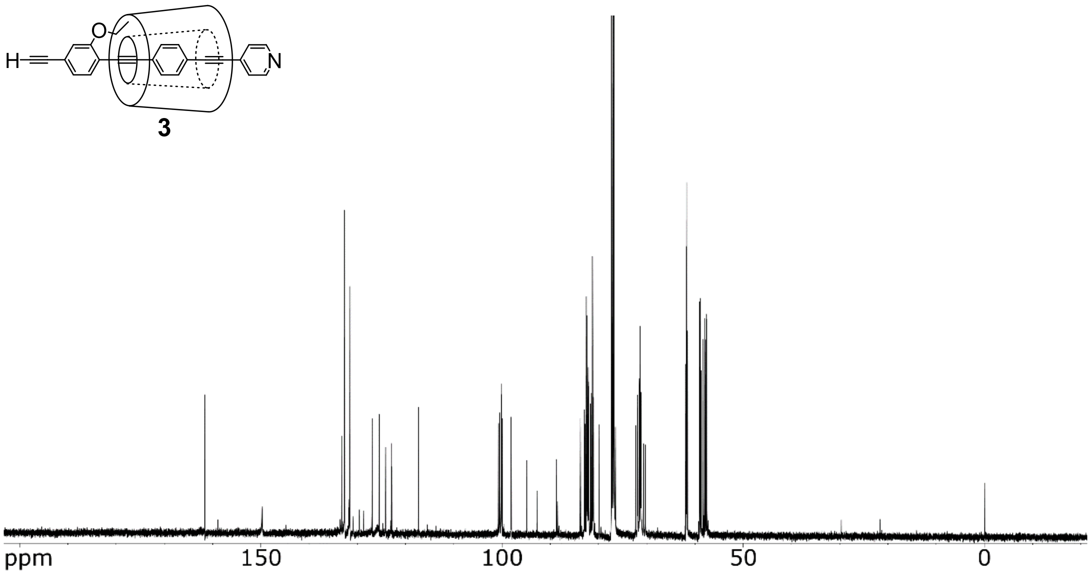

Supplementary Figure 23 ^{13}C NMR spectrum (126 MHz, $CDCl_3$) of **3**

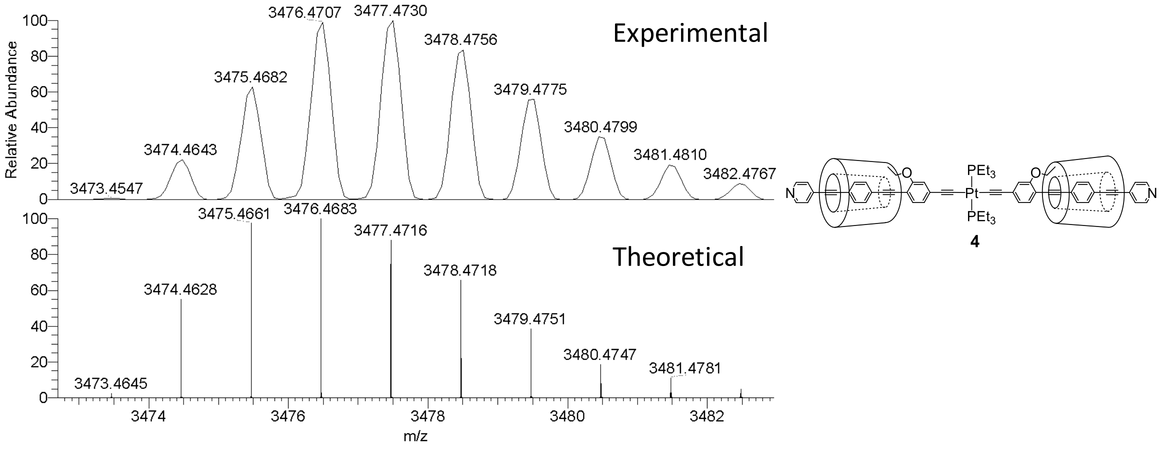

Supplementary Figure 24 MALDI TOF-MS spectrum of **4**

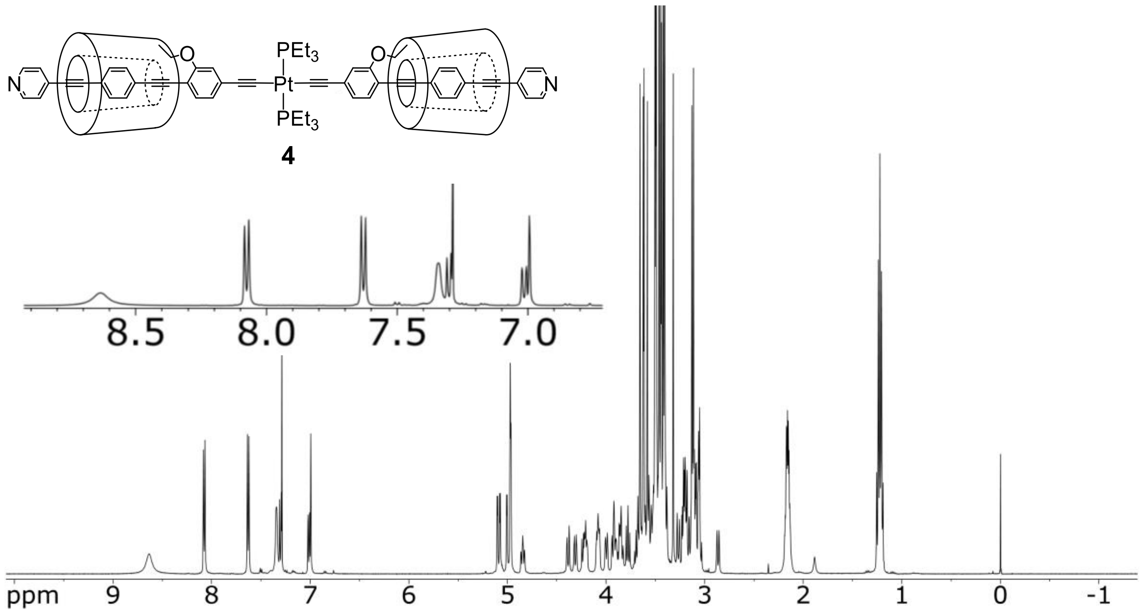

Supplementary Figure 25 ^{1}H NMR spectrum (500 MHz, $CDCl_3$) of **4**

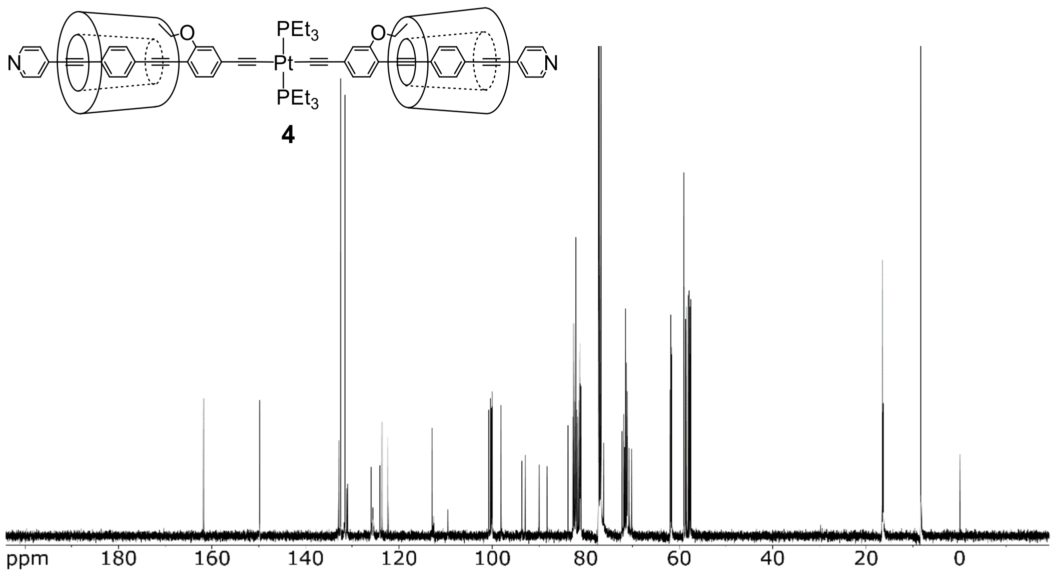

Supplementary Figure 26 ^{13}C NMR spectrum (126 MHz, $CDCl_3$) of **4**

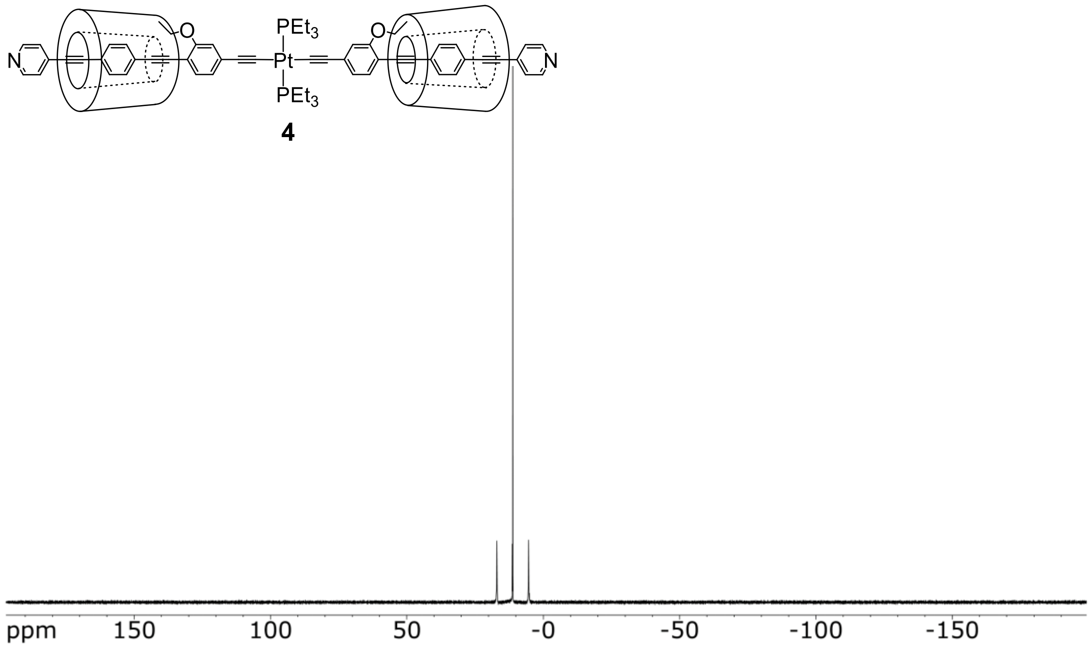

Supplementary Figure 27 ^{31}P NMR spectrum (202 MHz, $CDCl_3$) of **4**

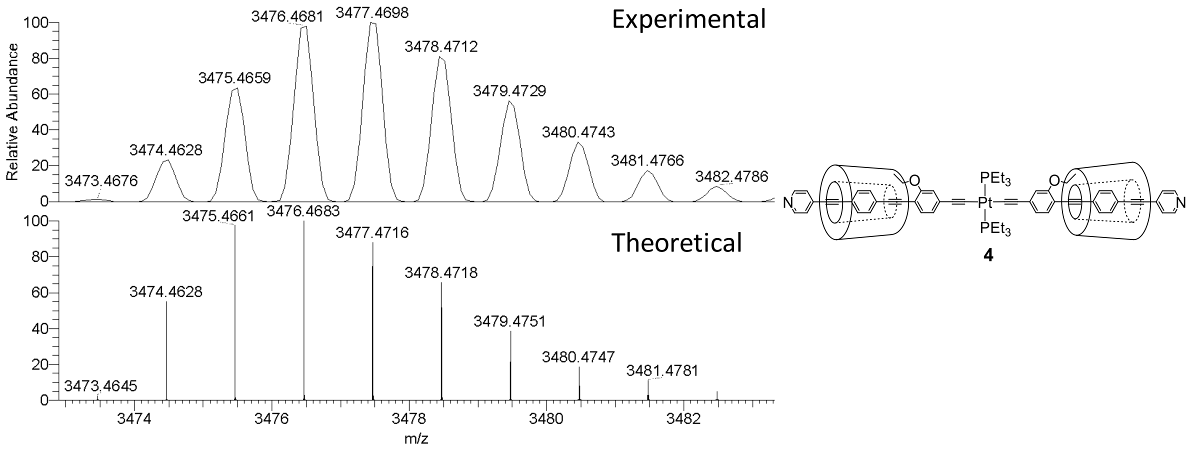

Supplementary Figure 28 MALDI TOF-MS spectrum of **4′**

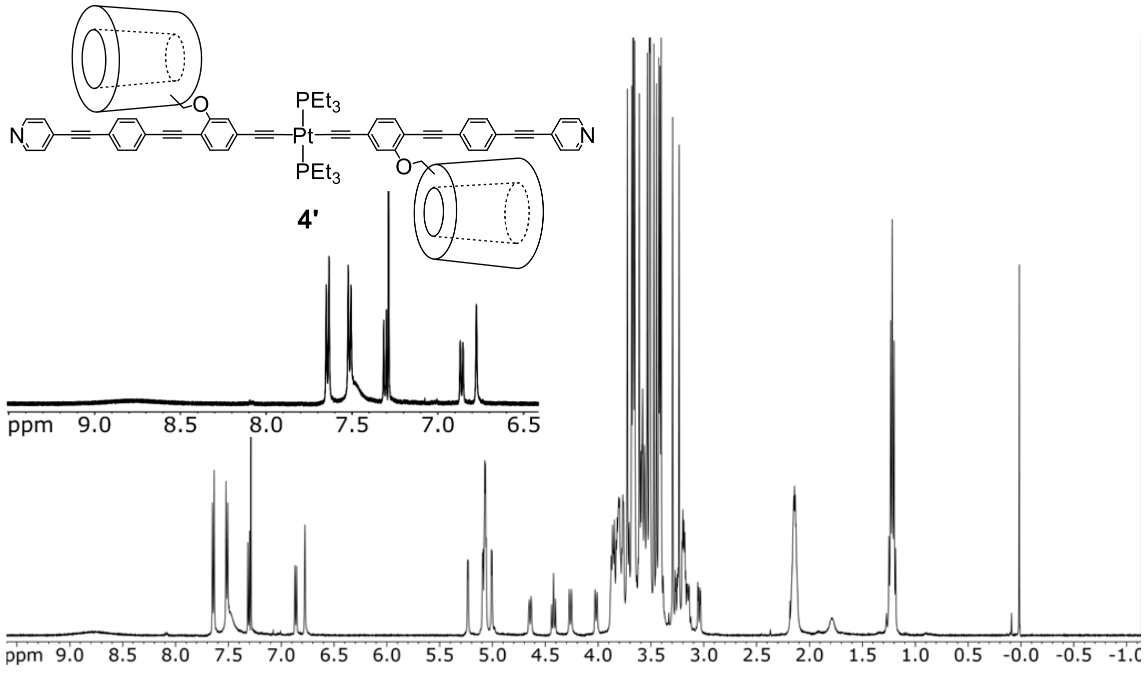

Supplementary Figure 29 ^{1}H NMR spectrum (500 MHz, $CDCl_3$) of **4′**

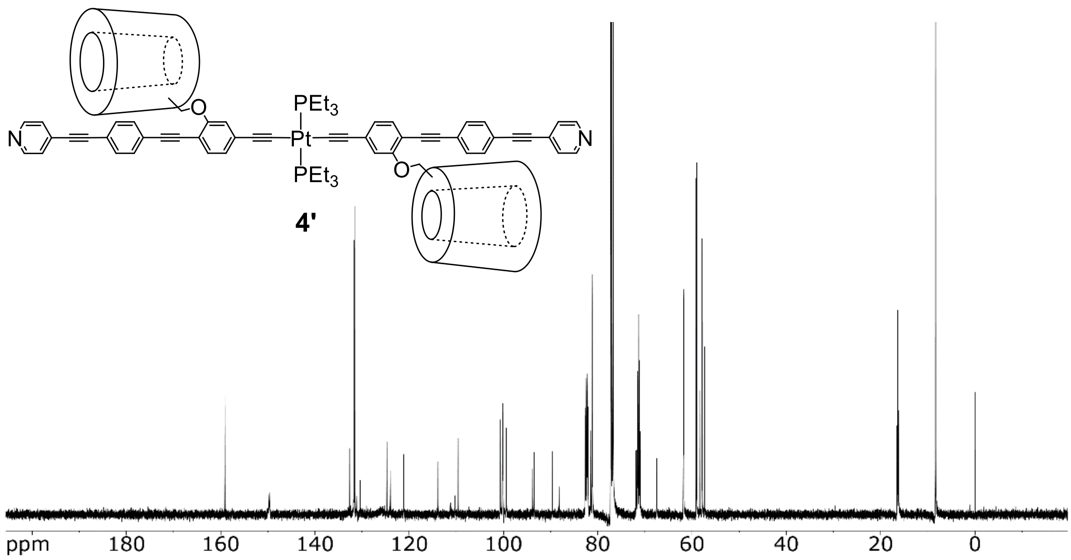

Supplementary Figure 30 ^{13}C NMR spectrum (126 MHz, $CDCl_3$) of **4′**

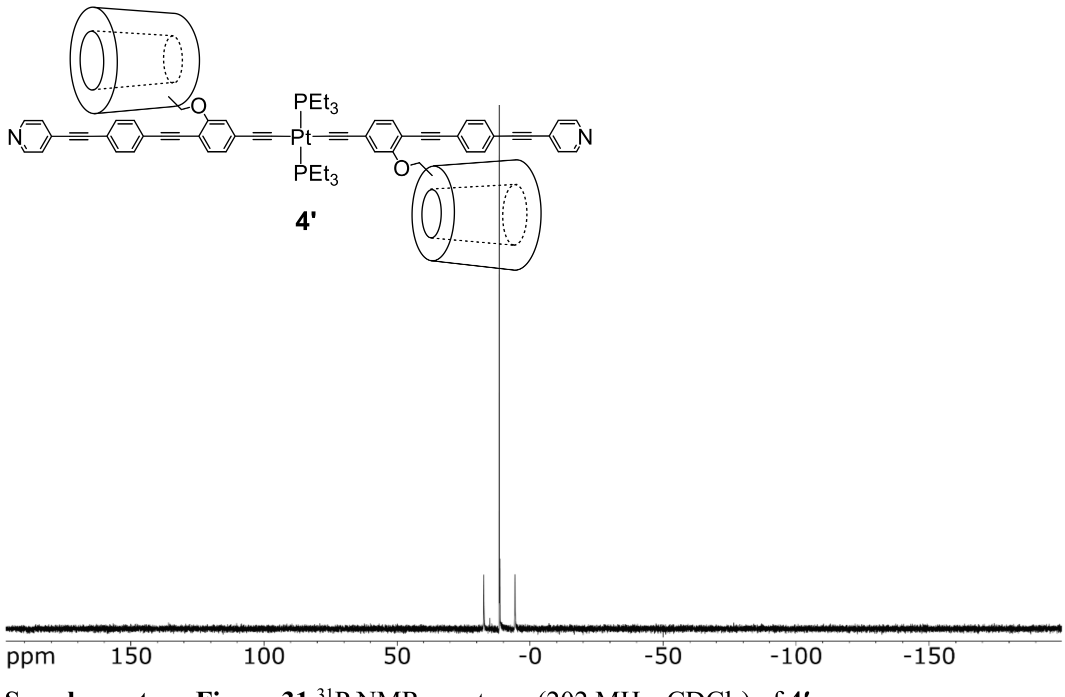

Supplementary Figure 31 ^{31}P NMR spectrum (202 MHz, $CDCl_3$) of **4′**

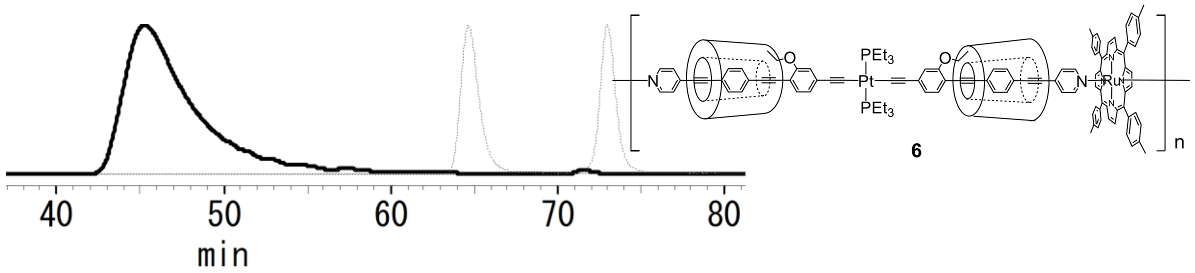

Supplementary Figure 32 SEC analyses (detected: UV 380 nm) of reaction mixture of **6** (solid) and each monomer **4** and **5** (dash)

^{1}H NMR (500 MHz, $CDCl_3$, r.t.) of **6**

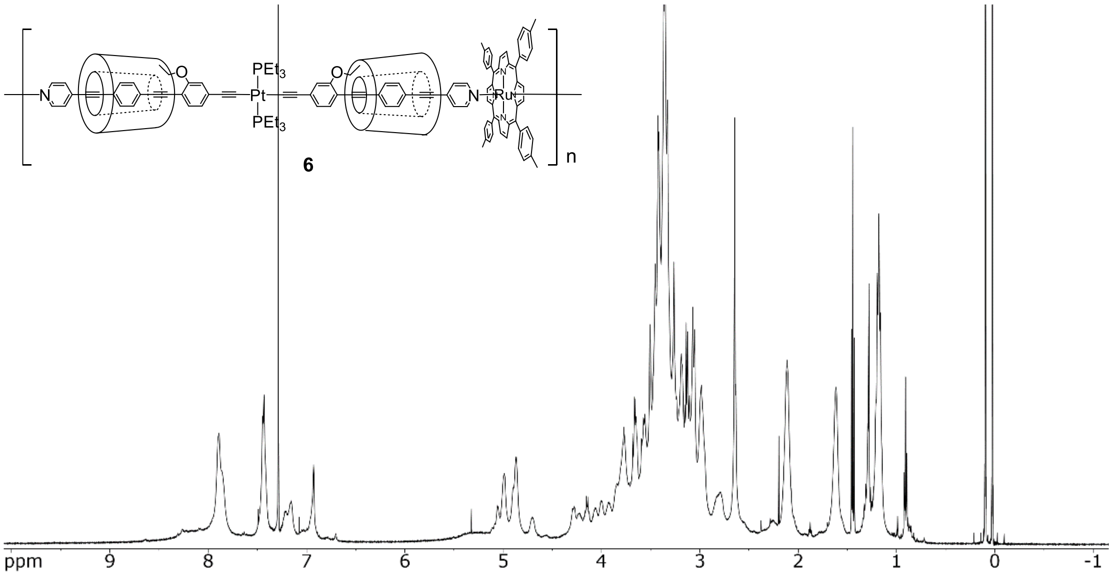

Supplementary Figure 33 ^{1}H NMR spectrum (500 MHz, $CDCl_3$) of **6**

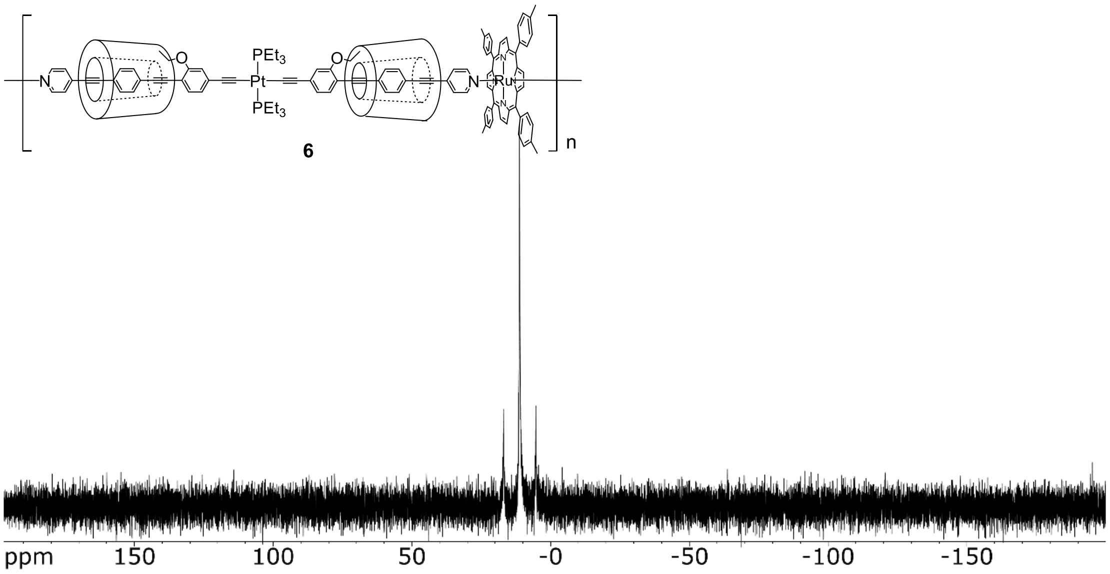

Supplementary Figure 34 ^{31}P NMR spectrum (202 MHz, $CDCl_3$) of **6**

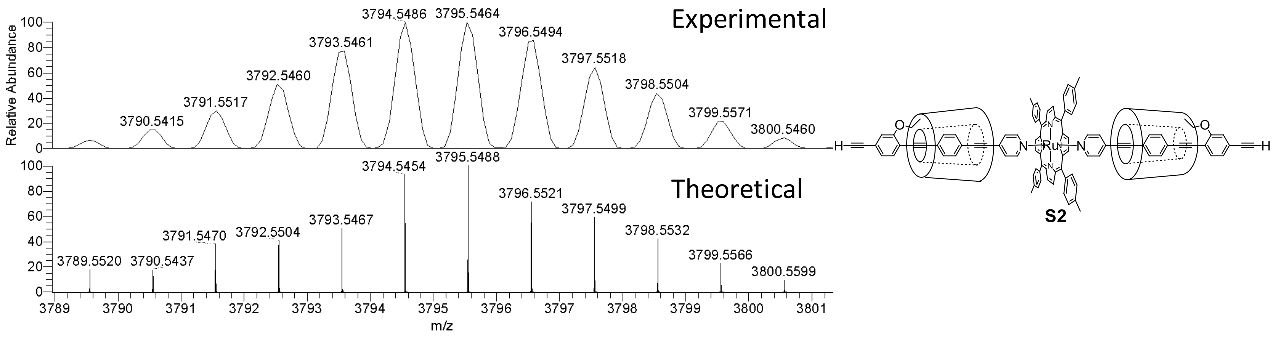

Supplementary Figure 35 MALDI TOF-MS spectrum of **S2**

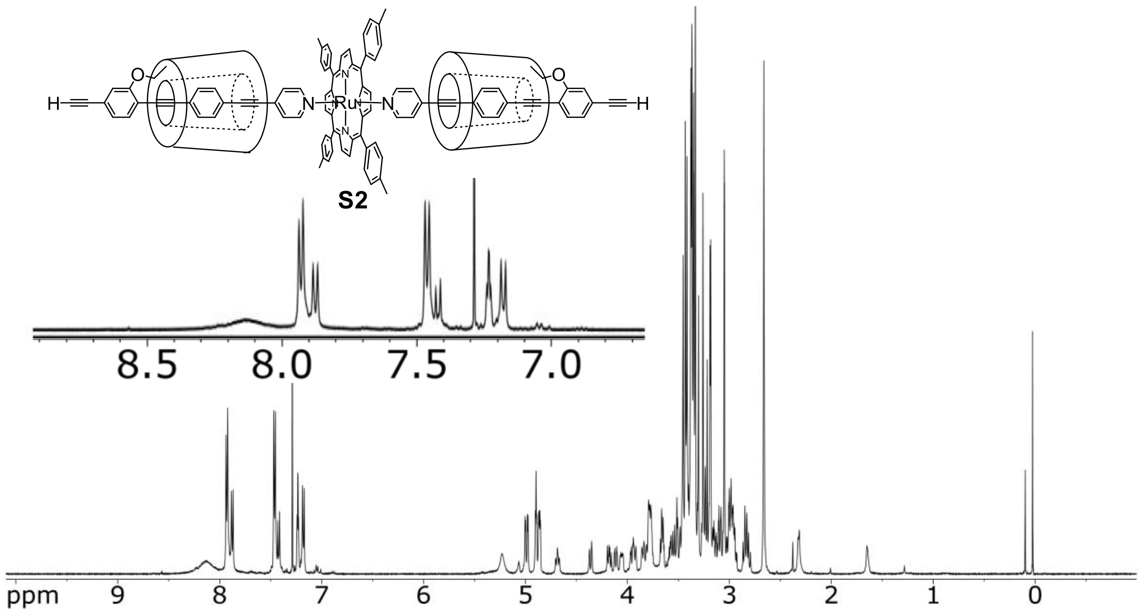

Supplementary Figure 36 ^{1}H NMR spectrum (500 MHz, $CDCl_3$) of **S2**

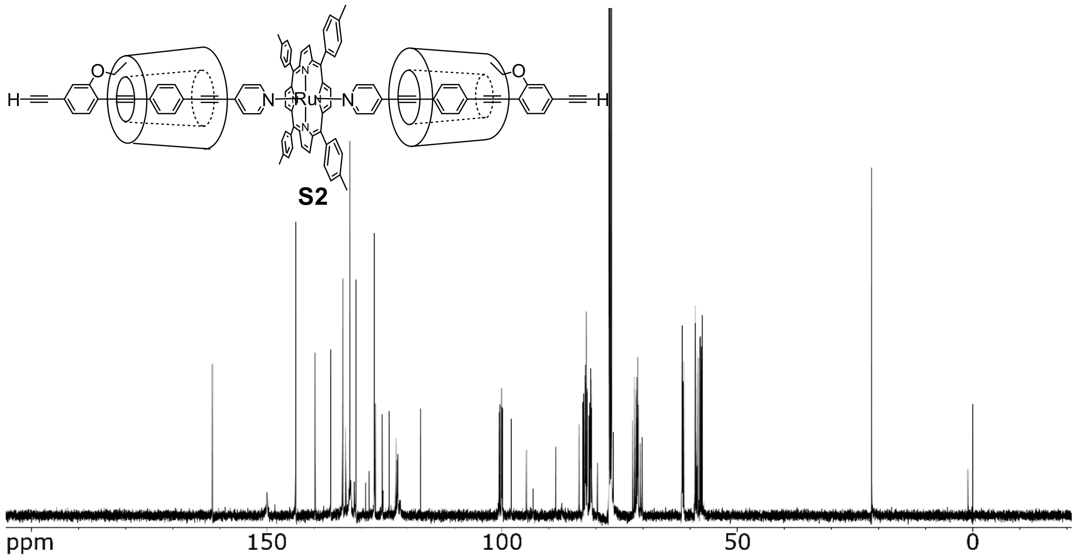

Supplementary Figure 37 ^{13}C NMR spectrum (126 MHz, $CDCl_3$) of **S2**

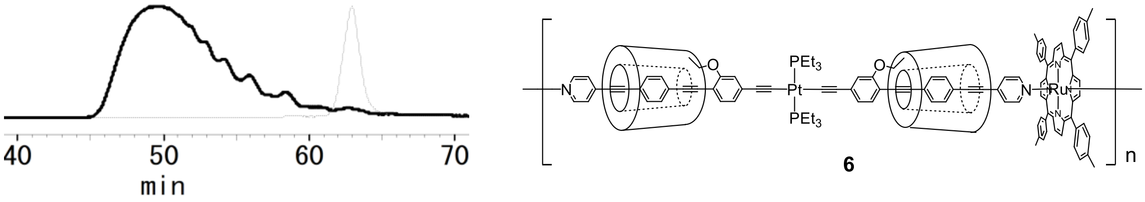

Supplementary Figure 38 SEC analyses (detected: UV 380 nm) of reaction mixture of **6** (solid) and monomer **S2** (dash)

Supplementary References

1. Pangborn, A. B., Giardello, M. a., Grubbs, R. H., Rosen, R. K. & Timmers, F. J. Safe and convenient procedure for solvent purification. *Organometallics* **15,** 1518–1520 (1996).
2. Masai, H., Terao, J., Fujihara, T. & Tsuji, Y. Rational design for rotaxane synthesis through intramolecular slippage: control of activation energy by rigid axle length. *Chem. - A Eur. J.* **22,** 6624–6630 (2016).
3. Masai, H. *et al.* Synthesis of one-dimensional metal-containing insulated molecular wire with versatile properties directed toward molecular electronics materials. *J. Am. Chem. Soc.* **136,** 1742–1745 (2014).
4. Maseras, F. & Morokuma, K. IMOMM: A new integrated ab initio + molecular mechanics geometry optimization scheme of equilibrium structures and transition states. *J. Comput. Chem.* **16,** 1170–1179 (1995).
5. Humbel, S., Sieber, S. & Morokuma, K. The IMOMO method: integration of different levels of molecular orbital approximations for geometry optimization of large systems: test for *n*-butane conformation and S_N2 reaction: $RCl+Cl^-$. *J. Chem. Phys.* **105,** 1959–1967 (1996).
6. Svensson, M. *et al.* ONIOM: A multilayered integrated MO + MM method for geometry optimizations and single point energy predictions. A test for diels−alder reactions and $Pt(Pt\text{-}Bu)_3)_2$ + H_2 oxidative addition. *J. Phys. Chem.* **100,** 19357–19363 (1996).
7. M. J. Frisch, *et al.* Gaussian09; Revision C.01; Gaussian, Inc.; Wallingford CT; 2010.
